# In‐Doped ZnO Electron Transport Layer for High‐Efficiency Ultrathin Flexible Organic Solar Cells

**DOI:** 10.1002/advs.202402158

**Published:** 2024-06-25

**Authors:** Xiujun Liu, Yitong Ji, Zezhou Xia, Dongyang Zhang, Yingying Cheng, Xiangda Liu, Xiaojie Ren, Xiaotong Liu, Haoran Huang, Yanqing Zhu, Xueyuan Yang, Xiaobin Liao, Long Ren, Wenliang Tan, Zhi Jiang, Jianfeng Lu, Christopher McNeill, Wenchao Huang

**Affiliations:** ^1^ State Key Laboratory of Advanced Technology for Materials Synthesis and Processing School of Materials Science and Engineering Wuhan University of Technology Wuhan 430070 P. R. China; ^2^ State Key Laboratory of Silicate Materials for Architectures Wuhan University of Technology Wuhan 430070 P. R. China; ^3^ International School of Materials Science and Engineering Wuhan University of Technology Wuhan 430070 P. R. China; ^4^ Australian Synchrotron Australian Nuclear Science and Technology Organisation (ANSTO) Clayton Victoria 3168 Australia; ^5^ School of Integrated Circuits Harbin Institute of Technology (Shenzhen) Shenzhen 518055 P. R. China; ^6^ School of Materials Science and Engineering Monash University Clayton Victoria 3168 Australia

**Keywords:** electron transport layer, indium doped zinc oxide, inverted organic solar cells, low‐temperature annealing, ultrathin flexible devices

## Abstract

Sol–gel processed zinc oxide (ZnO) is one of the most widely used electron transport layers (ETLs) in inverted organic solar cells (OSCs). The high annealing temperature (≈200 °C) required for sintering to ensure a high electron mobility however results in severe damage to flexible substrates. Thus, flexible organic solar cells based on sol–gel processed ZnO exhibit significantly lower efficiency than rigid devices. In this paper, an indium‐doping approach is developed to improve the optoelectronic properties of ZnO layers and reduce the required annealing temperature. Inverted OSCs based on In‐doped ZnO (IZO) exhibit a higher efficiency than those based on ZnO for a range of different active layer systems. For the PM6:L8‐BO system, the efficiency increases from 17.0% for the pristine ZnO‐based device to 17.8% for the IZO‐based device. The IZO‐based device with an active layer of PM6:L8‐BO:BTP‐eC9 exhibits an even higher efficiency of up to 18.1%. In addition, a 1.2‐micrometer‐thick inverted ultrathin flexible organic solar cell is fabricated based on the IZO ETL that achieves an efficiency of 17.0% with a power‐per‐weight ratio of 40.4 W g^−1^, which is one of the highest efficiency for ultrathin (less than 10 micrometers) flexible organic solar cells.

## Graphical abstract

An indium‐doped zinc oxide (IZO) electron transport layer (ETL) is developed for high‐efficiency inverted organic solar cells, and indium doping can improve electron extraction and suppress charge recombination. A 1.2‐micrometer‐thick ultrathin flexible OSC achieves an efficiency of 17.0% with a power‐per‐weight ratio of 40.4 W g^−1^, which is one of the highest values among ultrathin flexible organic solar cells.

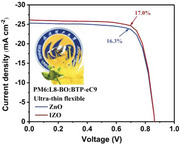

## 1 Introduction

Organic solar cells (OSCs) with bulk heterojunction (BHJ) structure composed of donor and acceptor have gained much attention in the last three decades due to their lightweight, good mechanical properties, and rapid energy payback time.^[^ ^1^, ^2^, ^3^, ^4^ ^]^ Through the development of new active materials and the optimization of device structures and morphology, the power conversion efficiency (PCE) of rigid single‐junction OSCs has now exceeded 19%.^[^ ^4^, ^5^, ^6^, ^7^ ^]^ However, the efficiency of flexible OSCs still lags far behind that of rigid counterparts.^[^ ^8^ ^]^ Flexible OSCs still need to improve both efficiency and stability, which are two key factors hindering the commercialization of this emerging flexible photovoltaic technology.^[^ ^9^, ^10^ ^]^

The inverted architecture is widely used in organic solar cells because it exhibits better stability and facilitates superior vertical phase separation of the active layer.^[^ ^11^, ^12^ ^]^ In the inverted architecture, the electron transport layer (ETL) plays an important role in determining device performance because it not only enhances charge carrier extraction but also affects the morphology of the active layer.^[^ ^12^, ^13^ ^]^ Existing ETL materials are generally classified into three types: polymers (PFN, PEI, and PEIE),^[^ ^14^, ^15^ ^]^ organic small molecules (PDINN and PDINO),^[^ ^16^, ^17^ ^]^ and inorganic interface materials (ZnO, TiO_x_, and SnO_x_).^[^ ^18^, ^19^, ^20^, ^21^ ^]^ Among these ETLs, ZnO is a commonly used inorganic interfacial material due to its good stability, proper energy level, and high transparency in the visible region.^[^ ^16^, ^17^, ^18^, ^19^, ^20^ ^]^ Conventional ZnO film can be easily prepared by the sol–gel method. However, it requires relatively high annealing temperatures (above 200 °C) to promote the chemical reaction and improve ZnO crystallization. Annealing at such high temperatures can damage flexible substrates such as PET and PEN. Thus, flexible devices based on sol–gel processed ZnO exhibit inferior efficiency compared to their rigid counterparts.^[^ ^22^, ^23^, ^24^ ^]^ Zhou et al. demonstrated that sol–gel processed ZnO films prepared at a low temperature exhibit a high density of defects, which act as recombination sites at the interface, which negatively affect device performance and stability.^[^ ^25^, ^26^, ^27^ ^]^

Several strategies have been proposed to eliminate the effects of defects in sol–gel processed ZnO film, including surface modification^[^ ^28^ ^]^ and doping passivation.^[^ ^10^ ^]^ Chen et al. developed a small molecule ((2‐(3‐(dimethylamino) propyl)‐1,3‐dioxo‐2,3‐dihydro‐1*H*‐benzo[*de*]isoquinoline‐6,7‐dicarboxylic acid), deposited on the top of ZnO film to passivate surface defects. Thus, the device's efficiency with the passivation layer was improved to 18.2%.^[^ ^29^ ^]^ In addition, PEIE is another popular dipole molecule used to modify the ZnO surface and optimize the energy level of ETLs, significantly improving the device's efficiency.^[^ ^14^, ^30^ ^]^ Unfortunately, the thickness of modified small molecules is strictly limited to up to 5 nm. Thus, it is still a challenging issue in large‐area fabrication. The elemental doping strategy has been recognized as an alternative method to modify the optical and electronic properties of ZnO ETLs. The electrical properties of ZnO have been improved by various doping elements with different valence states, such as lithium (Li), aluminum (Al), and zirconium (Zr).^[^ ^10^, ^20^, ^31^, ^32^ ^]^ Doping with such elements increases the concentration of carriers in ZnO film and improves electrical conductivity. The choice of element is important, with a suitable ionic radius required to support a high electron mobility. The efficiency of flexible devices based on these doped ZnO films still requires improvement to match devices based on rigid substrates. This motivates the need to develop new doping strategies.

According to the periodic table, indium ions (In^3+^) (0.81 Å) have a similar ionic radius to zinc ions (Zn^2+^) (0.74 Å), with In_2_O_3_ also having a high electron mobility (≈25 cm^2^ V^−1^ s^−1^) and high transmittance in the visible region.^[^ ^19^, ^33^ ^]^ In this work, we employ sol–gel‐processed ETLs based on In‐doped ZnO (IZO) in organic solar cells. Replacing Zn^2+^ with In^3+^ as a shallow donor results in the generation of extra free electrons, leading to an increased carrier concentration and mobility. It is shown that In‐doped ETLs not only improve charge extraction but also significantly suppress the generation of oxygen defects in ZnO processed with a low‐temperature annealing, which can reduce trap‐assisted charge combination. The efficiency of OSCs based on a PM6:L8‐BO binary blend increases from 17.0% in the pristine ZnO‐based device to 17.8% in the IZO‐based device. A champion efficiency of 18.1% can be achieved in PM6:L8‐BO:BTP‐eC9 ternary solar cells by using IZO as the ETL, which is one of the highest efficiency for the inverted OSCs. In addition, after doping with indium, the annealing temperature can be decreased to 140 °C, which is suitable for flexible substrate. An ultrathin flexible device with a total thickness of 1.2 µm is also developed using IZO ETL, achieving an efficiency of 17.0%. Such ultrathin flexible OSCs furthermore exhibit good mechanical stability, retaining 83% of their original efficiency after a 100‐cycle stretching/compression tests.

## 2 Results and Discussion

**Figure 1:**
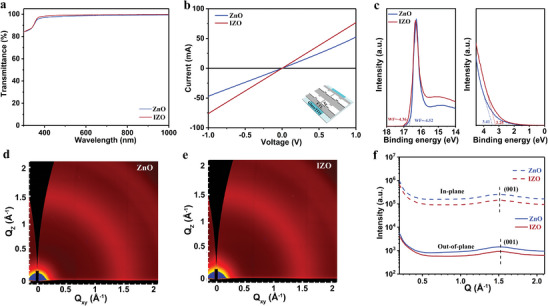
a) UV–vis spectra of ZnO and IZO films. b) Electrical conductivity of ZnO and IZO films. c) UPS spectra of ZnO and IZO films. Grazing incidence wide‐angle X‐ray scattering (GIWAXS) patterns of d) ZnO and e) IZO films. f) Line scattering curves cut from 2D GIWAXS patterns along out‐of‐plane (OOP) and in‐plane (IP) directions.

The transmittance spectra of ZnO and IZO film with the same thickness are compared in **Figure** **1**a and S1 (Supporting Information). It is observed that the IZO film exhibits slightly higher transmittance in both visible and NIR regions compared to the ZnO film. This characteristic can facilitate increased light absorption in active layers, resulting in higher photocurrent generation. In order to further investigate the effects of indium doping on the electrical properties of ZnO, the electrical conductivities (*σ*_0_) of both ZnO and IZO film are measured via *J–V* characteristic curves at 140 °C with a device structure of ITO/ETL/Ag, as illustrated in Figure 1b. Using an indium concentration of 5% (which results in the best device efficiency, see Figure S11 and Table S3, Supporting Information), the electrical conductivity is found to increase from 1.39 × 10^−4^ S m^−1^ for the pristine ZnO‐based film to 2.22 × 10^−4^ S m^−1^ for the IZO‐based film, indicating that indium doping enhances the electrical conductivity of ZnO. The conductivity of IZO thin films with different indium concentrations is shown in Figure S2 and Table S1 (Supporting Information). It is observed that the conductivity initially increases as a function of indium doping content. However, In‐doping up to 9%, the film's conductivity reaches a peak value, followed by decreasing with the increased indium doping content, which is caused by the aggregation of excessive doping elements at the grain boundaries, adversely affecting the film's conductivity. In addition, we investigate the conductivity changes of pure ZnO different annealing temperatures from 100 to 500 °C (Figure S3, Supporting Information), which reveals the significant effects of the appropriate amount of doping and suitable annealing temperatures on the enhancement of film conductivity.

Surface work function (WF) measurements are conducted on ZnO and IZO films, as depicted in Figure 1c.^[^ ^34^ ^]^ The ZnO film exhibits an estimated WF of −4.52 eV, while the WF of the IZO film is −4.36 eV. This suggests that appropriate doping of indium can modify the energy level of ZnO, facilitating electron collection and transport properties by reducing the energy level difference between the active layer and ETL. The optical bandgap values of ZnO and IZO were calculated by plotting *(αhv)^2^* versus *hv*, as shown in Figures S4 and S5 (Supporting Information). The calculated bandgaps were 3.45 and 3.41 eV, respectively. The doping of indium exhibits negligible effects on the bandgap of ETL film.^[^ ^35^ ^]^ The crystallization behavior of ZnO and IZO film is further investigated using synchrotron‐based grazing incidence wide‐angle X‐ray scattering (GIWAXS). The 2D scattering patterns of ZnO and IZO film exhibited a similar scattering peak at *q* = 1.5 Å^−1^, corresponding to the (001) crystal plane^[^ ^19^ ^]^; see Figure 1d,e. As shown in Figure 1f and Table S2 (Supporting Information), the coherence length (CCL) of the (001) peak, calculated from the Scherrer equation, remains unchanged after the doping of indium in ZnO film. ZnO and IZO films exhibit similar crystallinity.

**Figure 2:**
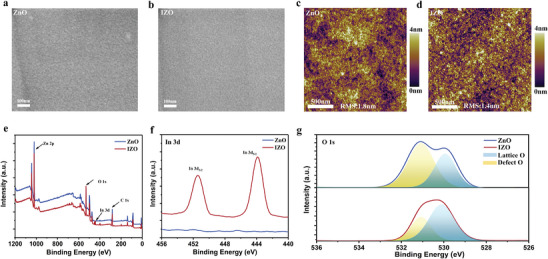
SEM images of a) ZnO film and b) IZO film. AFM images of c) ZnO film and d) IZO film. e) XPS survey spectra of ZnO and IZO films. f) In 3d and g) O 1s spectra of ZnO and IZO films.

Surface morphologies of the ZnO and IZO films are examined using scanning electron microscopy (SEM) and atomic force microscopy (AFM) (**Figure** **2**a–d). It can be observed that the IZO film exhibits a denser surface compared to ZnO. In this study, ZnO and IZO film samples are prepared on silicon wafers and tested for root mean square roughness (RMS) with the help of AFM. The results showed that the RMS of the silicon wafer substrate is 0.3 nm (Figure S7, Supporting Information). In comparison, the RMS of ZnO and IZO films prepared by the spin‐coating method are 1.8 and 1.4 nm, respectively.

X‐ray photoelectron spectroscopy (XPS) is used to analyze the change of the surface chemical bonding induced by In‐doping. The XPS survey (Figures 2e,f) shows an indium signal (at 444.0 and 452.0 eV) in the IZO film, indicating the successful incorporation of indium. Zn 2p and O 1s XPS spectra were acquired to analyze the defects in the ZnO film. The Zn 2p peaks after indium doping show a 0.6 eV shift toward the lower binding energy (Figure S8, Supporting Information). This shift may be caused by the substitution of Zn, resulting in the sharing of more electrons with the O atom.

Electronic structure analysis of ZnO and IZO crystals was performed using spin‐polarized density‐functional theory (DFT) as shown in Figure S9 (Supporting Information). The computational results show that the loss of electrons of Zn atoms in In‐doped IZO is reduced compared to that of pure ZnO, the conclusion is consistent with the decrease in the binding energy of the Zn 2p orbitals detected by X‐ray photoelectron spectroscopy (XPS) experiments. The O 1s spectra (Figure 2g) can be fitted with two peaks: a peak located at ≈530.0 eV corresponding to lattice oxygen, and a peak at 531.0 eV is associated with oxygen defects (oxygen vacancies (*V*_o_)).^[^ ^36^ ^]^ It is noted that the intensity of the peak at 531.0 eV in the IZO film is weakened compared to that of the ZnO film, which implies a significant decrease in the concentration of defective states in the IZO film. The formation energies of vacancy oxygen in ZnO and IZO film are calculated using density functional theory (DFT) (Figure S10, Supporting Information). The calculation results show that the oxygen vacancy formation energy is 2.5 eV for ZnO and 3.5 eV for IZO, indicating that indium doping makes the formation of oxygen vacancies more difficult and suppresses the formation of oxygen defects.

**Figure 3:**
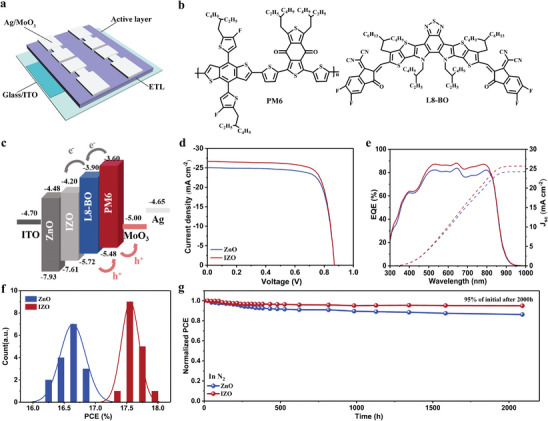
a) Device structure of inverted organic solar cells. b) Molecular structure of active layer materials. c) Energy level diagram of inverted organic solar cells. d) *J–V* characteristics of organic solar cells with different electron transport layers under AM1.5G illumination at 100 mW cm^−2^. e) EQE curves of organic solar cells with different electron transport layers. f) Statistics of PCE values of organic solar cells with different electron transport layers. g) Stability of organic solar cells with different electron transport layers.

OSCs are fabricated with an inverted structure of ITO/ZnO(IZO)/active layer/MoO_3_/Ag, as shown in **Figure** **3**a. The chemical structures of PM6:L8‐BO are shown in Figure 3b. Figure S11 (Supporting Information) shows the devices doped with different indium contents. By comparison, it reveals improvements in the short circuit current density (*J*_sc_) and fill factor (FF) with increasing indium content. Particularly, the devices achieved the highest efficiency at an indium content of 5%. In addition, a significant increase in the external quantum efficiency (EQE) profiles was observed. Specific data summarizing these findings can be found in Table S3 (Supporting Information). Therefore, use pristine ZnO and 5% In‐doped ZnO for comparison. The thickness IZO in the optimal preparation procedure is 21 nm, as shown in Figure S12 and Table S4 (Supporting Information). ZnO and IZO films measured under the same preparation conditions have the same thickness (Figure S13, Supporting Information). The optimal preparation procedure has an annealing temperature of 140 °C for IZO and 200 °C for ZnO (Figures S14 and S15, Supporting Information) and the specific performance parameters of the devices are summarized in Tables S5 and S6 (Supporting Information). The particular preparation flow chart is shown in Figure S17 (Supporting Information).

The energy level of each layer in the device is illustrated based on the measured WF (work function) (Figure 3c). Better alignment between the IZO and L8‐BO energy levels is achieved. Current density and voltage (*J–V*) curves and external quantum efficiency (EQE) characteristics of the corresponding devices are shown in Figure 3d,e. The related specific parameters are summarized in **Table** **1**. Devices based on the ZnO ETL show an average power conversion efficiency (PCE) of 16.6% with an open circuit voltage (*V*_oc_) of 0.87 V, a short circuit current density (*J*_sc_) of 24.9 mA cm^−2^, and a fill factor (FF) of 76.7%. When IZO is used as ETL, *J*_sc_ increases to 26.5 mA cm^−2^and PCE increases to 17.5%. The bar distribution chart of PCE for ZnO‐based and IZO‐based devices is shown in Figure 3f, which shows the extraction of 16 identical devices from different batches. The average PCEs for ZnO and IZO‐based devices are 16.6% ± 0.3% and 17.5% ± 0.3%, respectively, indicating that In‐doping is an effective strategy to improve PV performance. External quantum efficiency (EQE) measurements further confirm the enhanced photocurrent response in IZO‐based devices compared to ZnO‐based ones (Figure 3e). The EQE in the visible region significantly improves for the IZO‐based devices. The values from the EQE‐integrated *J*_sc_ are summarized in Table 1, which are consistent with *J*_sc_ measured by *J–V* with a deviation of less than 5%.

**Table 1:** The parameter of PM6:L8‐BO OSCs with different electron transport layers.

ETL/Active layer ^a)^	*V*_oc_ [V]	*J*_sc_ [mA cm^−2^]	Calc. *J*_sc_ [mA cm^−2^]^b)^	FF [%]	PCE [%]
ZnO/PM6:L8‐BO	0.875 (0.870±0.005)	25.05 (24.94±0.11)	24.20	77.50 (76.73±0.77)	16.97 (16.66±0.31)
IZO/PM6:L8‐BO	0.870 (0.867±0.003)	26.71 (26.55±0.16)	25.66	76.95 (76.19±0.76)	17.81 (17.55±0.26)

^a)^ The average results from 16 devices;

^b)^ Calculated current densities from EQE curves.

The stability of the devices based on different ETLs is also assessed. After more than 2000 h in an inert environment, IZO‐based devices retained 95% of their original efficiency, while ZnO‐based devices retained 87% (Figure 3g). The IZO‐based devices also demonstrate better stability than the ZnO‐based devices under thermal stress and light illumination (Figures S18–S20, Supporting Information). Stability results show that the IZO‐based OSCs have a higher operational stability, and their *T_80_* lifetimes are significantly enhanced from 156 to 265 h as compared with the ZnO‐based OSCs. The XPS analysis shows that In‐doped ZnO effectively reduces the concentration of oxygen defects. Oxygen defects tend to form and intensify on the ZnO surface, leading to an increase in traps and affecting the charge collection efficiency.^[^ ^37^ ^]^ From the TPC decay curves in Figure S20c,d (Supporting Information), it can be seen that the charge extraction efficiency of ZnO‐based cells is significantly reduced under light and thermal aging conditions. In contrast, the IZO ETL device significantly suppresses the growth of subsequent oxygen vacancies, thus enhancing the overall stability.^[^ ^38^ ^]^ Overall, the In‐doping process can effectively improve the photovoltaic performance and stability of the OSCs.

**Figure 4:**
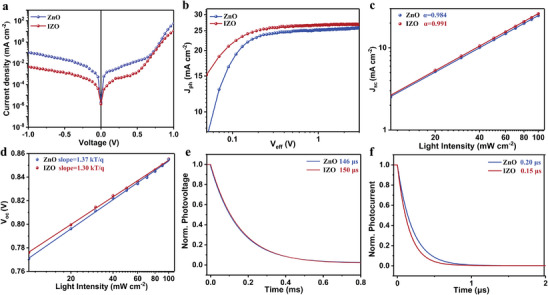
a) *J–V* characteristic curves of the devices with different cathode interlayers in dark conditions. b) Photocurrent density (*J*_ph_) versus effective voltage (*V*_eff_) curves of the devices. c) Dependence of *J*_sc_ on light intensity. d) Dependence of *V*_oc_ on light intensity. e) Transient photovoltage (TPV) curves of organic solar cells with different electron transport layers. f) Transient photocurrent (TPC) curves of organic solar cells with different electron transport layers.

**Figure** **4**a shows the results of dark current measurements of the devices under dark conditions. It is observed that the reverse saturation current of the IZO‐based device is significantly suppressed compared to the ZnO‐based device. This suppression suggests that the doping of In in the ETL can enhance the charge selection and transport properties. To analyze the charge collection characteristics of ZnO and IZO‐based OSCs, the photocurrent density (*J*_ph_) as a function of effective voltage (*V*_eff_) is measured. *J*_ph_ is determined as the difference between the photocurrent density under illumination (*J*_L_) and the dark current density (*J*_D_). *V*_eff_ is the difference between the voltage at *J*_ph_ = 0 (*V*_0_) and the applied external bias voltage (*V*_bias_). The exciton dissociation efficiency (*P*_diss_) is calculated by dividing *J*_ph_ by the saturation current density (*J*_sat_).^[^ ^39^ ^]^ According to Figure 4b and Table S7 (Supporting Information), the *P*_diss_ values for ZnO and IZO‐based devices are 95.2% and 98.2%, respectively. The higher *P*_diss_ in IZO‐based devices suggests more efficient exciton dissociation. Moreover, the higher *P*_coll_ is also obtained in IZO‐based devices, indicating improved charge collection compared to ZnO‐based devices. The improvement could be partially attributed to the increased built‐in electric field resulting from the shift of the work function (WF) of the IZO film and better charge extraction. Under the same preparation conditions, the micromorphology of the active layer on ZnO and IZO substrates was analyzed by GIWAXS data (Figures S21 and S22, Table S8, Supporting Information). PM6:L8‐BO adopts face‐on orientation on both ZnO and IZO. The (100) diffraction peak position in the In‐plane direction is almost the same, ≈0.30 Å^−1^ (*d* spacing: 20.94 Å), *π‐* *π* stacking (010). The diffraction peak is located near 1.73 Å^−1^ (*d* spacing: 3.63 Å) in the out‐of‐plane. However, the crystal coherence length (CCL) of the (010) diffraction peak and (100) diffraction peak of blends on the IZO ETL is slightly larger than those on the ZnO ETL, which has beneficial charge transport. These observations are consistent with the higher short‐circuit current density (*J*_sc_) in IZO‐based devices. The device physics of OSCs with different ETL materials is further characterized through measurements of *J*_sc_ and *V*_oc_ as a function of light intensity (*P*_light_). The relationship between *J*_sc_ and *P*_light_ in OSCs based on ZnO and IZO‐ETL is presented in Figure 4c. The device based on IZO‐ETL exhibits an α value of 0.991, closer to 1, indicating the reduced bimolecular recombination.

The dependence of *V*_oc_ on *P*_light_ is measured to investigate the trap‐assisted recombination mechanism. The slopes of ZnO‐ and IZO‐based devices are calculated to be 1.37 and 1.30 kT q^−1^, respectively (Figure 4d), indicating that charge trapping effects are suppressed in IZO‐based devices.^[^ ^40^, ^41^ ^]^ The difference in charge extraction dynamics in devices based on two different ETLs have been studied using transient photovoltage (TPV) and transient photocurrent (TPC); see Figure 4e,f. The decay time in the IZO‐based device (0.15 µs) is faster than the ZnO‐based counterparts, suggesting better charge extraction in the active layer/IZO interface in IZO ETL. Additionally, the charge recombination behavior of OSCs based on different ETLs is performed by transient photovoltage (TPV) measurements. The carrier lifetime (*τ*) is extracted from the fit of the TPV signal, with values of 146 and 150 µs in ZnO and IZO‐based devices, respectively. This observation indicates that slightly suppressed charge recombination is observed in the IZO‐based device.^[^ ^42^ ^]^ The *C*–*V* curves of ZnO and IZO ETL devices showed that IZO has a better built‐in electric field than ZnO devices (Figure S23, Supporting Information).^[^ ^43^ ^]^

To confirm that IZO is a universal ETL, OSCs with fullerene‐based blends (PTB7‐Th:PC_71_BM), non‐fullerene‐based blends (PM6:Y6 and PM6:L8‐BO:BTP‐eC9) and all‐polymer blends (PM6:PY‐IT) are investigated. The corresponding molecular structures and absorption spectra are shown in Figure S24 and S25 (Supporting Information). The photovoltaic performance in OSCs with different active layers is summarized in **Table** **2**. The IZO‐based OSCs with a ternary active layer of PM6:L8‐BO:BTP‐eC9 can achieve an efficiency of 18.1%, while the efficiency of ZnO‐based OSCs exhibits an efficiency of 17.2%. It can be concluded that OSCs utilizing IZO as the electron transport layer consistently exhibit better photovoltaic performance than pristine ZnO‐based devices (Figure S26, Supporting Information). Based on recently published results (Figure S27 and Table S10, Supporting Information), it can be verified that the In‐doping approach is a promising pathway to fabricate high‐efficiency inverted OSCs.

**Table 2:** The parameter of OSCs with different active layers and electron transport layers.

Active layer ^a)^	ETL	*V*_oc_ [V]	*J*_sc_ [mA cm^−2^]	Calc. *J*_sc_ [mA cm^−2^]^b)^	FF [%]	PCE [%]
PTB7‐Th:PC_71_BM	ZnO	0.797 (0.795 ± 0.002)	17.40 (17.23 ± 0.17)	16.54	69.30 (68.80 ± 0.50)	9.51 (9.45 ± 0.06)
	IZO	0.801 (0.797 ± 0.004)	17.97 (17.82 ± 0.15)	16.88	70.99 (70.26 ± 0.73)	10.24 (10.01 ± 0.23)
PM6:Y6	ZnO	0.830 (0.827 ± 0.003)	26.20 (26.03 ± 0.17)	25.14	72.04 (71.16 ± 0.88)	15.61 (15.33 ± 0.28)
	IZO	0.835 (0.830 ± 0.005)	26.99 (26.66 ± 0.33)	25.82	71.88 (71.34 ± 0.54)	16.15 (15.80 ± 0.35)
PM6:PY‐IT	ZnO	0.928 (0.925 ± 0.003)	23.40 (23.37 ± 0.03)	22.51	67.70 (67.42 ± 0.28)	14.68 (14.58 ± 0.10)
	IZO	0.931 (0.927 ± 0.004)	24.13 (24.02 ± 0.11)	23.14	67.43 (66.96 ± 0.47)	15.11 (14.92 ± 0.19)
PM6:L8‐BO:BTP‐eC9	ZnO	0.861 (0.857 ± 0.004)	26.10 (25.93 ± 0.17)	25.12	76.60 (75.84 ± 0.76)	17.18 (16.86 ± 0.32)
	IZO	0.859 (0.854 ± 0.005)	27.65 (27.53 ± 0.12)	26.74	76.54 (76.18 ± 0.36)	18.11 (17.92 ± 0.19)

^a)^ The average results from 12 devices;

^b)^ Calculated current densities from EQE curves.

**Figure 5:**
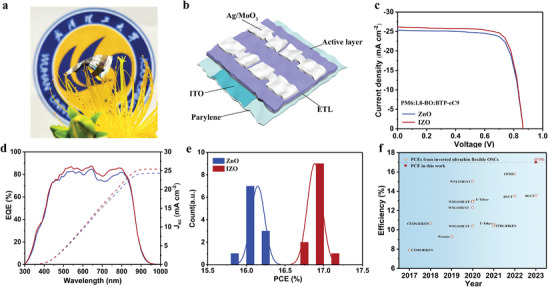
a) A photograph of inverted ultrathin flexible OSCs. b) Device structure of inverted ultrathin flexible OSCs. c) Inverted ultrathin flexible OSCs based on ZnO and IZO ETL *J–V* characteristic curves. d) EQE curves of the devices. e) Statistics of PCE values for different cathode interlayer devices. f) Summary chart of efficiency of inverted ultrathin flexible OSCs.

During the optimization of the annealing temperature of ZnO and IZO, it is observed that the efficiency of ZnO‐based devices shows an increase with increased temperature. OSCs based on ZnO ETL require an annealing temperature of over 200 °C to achieve the best efficiency. However, in devices with IZO‐based ETL, the optimized annealing temperature is reduced to 140 °C (Figures S14 and S15, Supporting Information). The specific photovoltaic parameters of OSCs as a function of annealing temperature are summarized in Tables S5 and S6 (Supporting Information). As shown in Figure S28 (Supporting Information), the parylene/ITO ultrathin flexible substrate cannot survive the high‐temperature treatment of over 200 °C. The lower annealing temperature of IZO‐ETL is advantageous for flexible devices. Consequently, ultrathin flexible OSCs with ZnO and IZO ETLs are fabricated on parylene/ITO substrates. The transmittance characteristics of parylene/ITO are depicted in Figure S29 (Supporting Information). **Figure** **5**a,b provides a photo and schematic of 1.2‐micrometer‐thick ultrathin flexible OSCs. A device structure of parylene/ITO/ZnO(IZO)/PM6:L8‐BO:BTP‐eC9/MoO_3_/Ag is used to fabricate ultrathin flexible OSCs. *J–V* curves of ultrathin flexible OSCs are shown in Figure 5c and summarized in **Table** **3**. Notably, the champion ultrathin OSC based on the IZO ETL exhibits a PCE of 17.0% with *V*_oc_ of 0.86 V, *J*_sc_ of 26.2 mA cm^−2^ and FF of 75.7%, and a power‐per‐weight ratio of 40.4 W g^−1^. In contrast, the efficiency of the ZnO‐based ultrathin flexible device is 16.3%, with *V*_oc_ of 0.86 V, *J*_sc_ of 25.2 mA cm^−2^, and FF of 74.6%. Figure 5e provides the PCE values statistics derived from 12 identical devices. The external quantum efficiency (EQE) spectra of ultrathin flexible devices are illustrated in Figure 5d. OSCs based on ZnO and IZO ETL exhibit integrated currents of 24.3 and 25.4 mA cm^−2^, which agree with the *J–V* curves. To the best of our knowledge, this result is one of the highest reported efficiencies for inverted ultrathin flexible devices (Figure 5f; Table S10, Supporting Information).

**Table 3:** The parameter of ultrathin flexible OSCs with different electron transport layers.

ETL/Active layer^a)^	*V*_oc_ [V]	*J*_sc_ [mA cm^−2^]	Calc. *J*_sc_ [mA cm^−2^]^b)^	FF [%]	PCE [%]
ZnO/PM6:L8‐BO:BTP‐eC9	0.861 (0.857 ± 0.004)	25.22 (25.19 ± 0.03)	24.30	75.20 (74.60 ± 0.60)	16.30 (16.13 ± 0.17)
IZO/PM6:L8‐BO:BTP‐eC9	0.861 (0.858 ± 0.003)	26.20 (26.15 ± 0.05)	25.41	75.73 (75.26 ± 0.47)	17.01 (16.90 ± 0.11)

^a)^ The average results from 12 devices;

^b)^ Calculated current densities from EQE curves.

**Figure 6:**
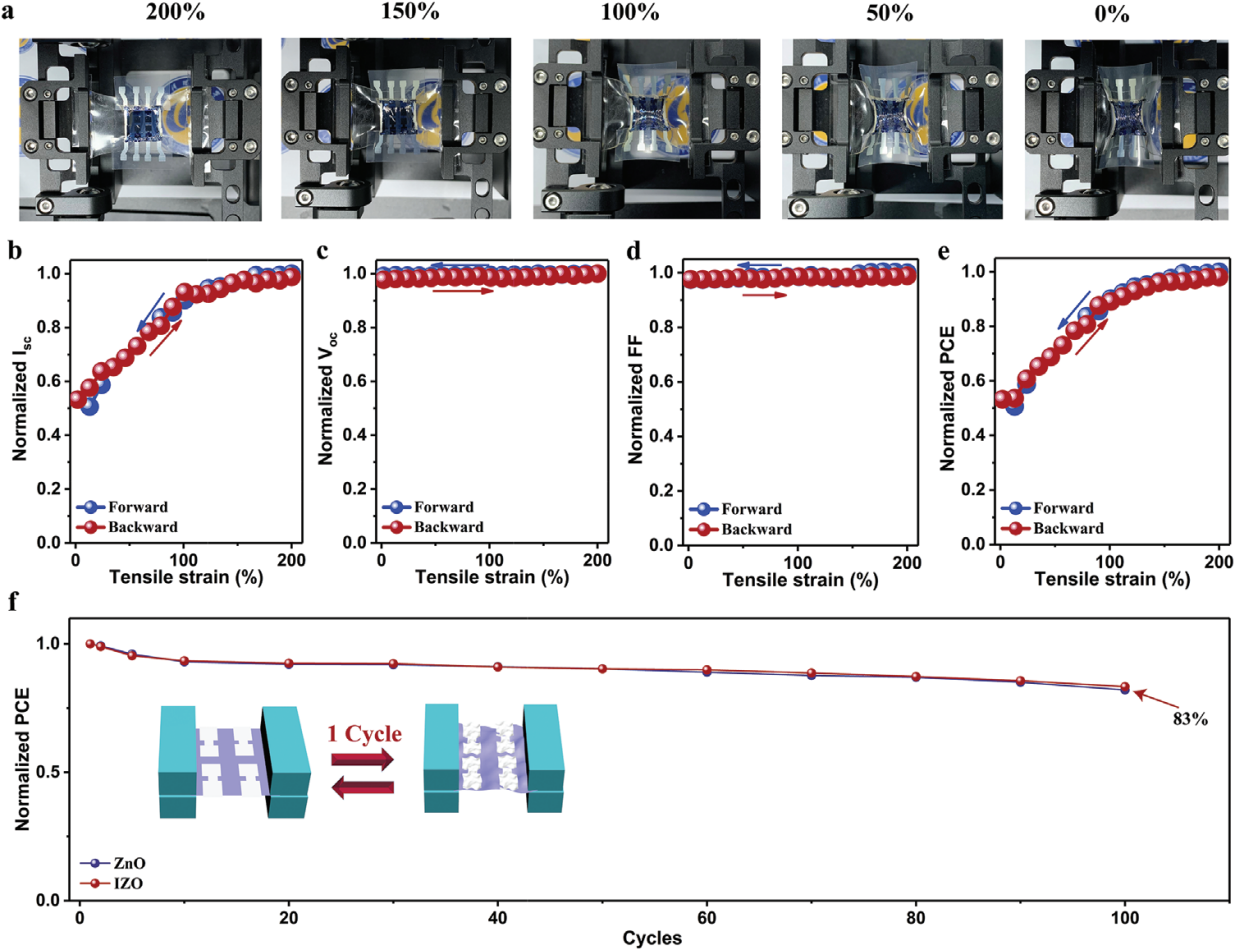
a) Images of ultrathin flexible inverted OSCs under stretching/compression test. The evolution of b) *I*_sc_. c) *V*_oc_. d) FF, and e) PCE as a function of compression rate. f) The mechanical stability of inverted ultrathin flexible OSCs under cyclic stretching/compression.

The mechanical stability of inverted ultrathin flexible OSCs based on ZnO and IZO ETL is also studied under cyclic stretching/compression testing, as shown in **Figure** **6**a. The ultrathin flexible OSCs are transferred to a pre‐stretched polymer elastomer. Subsequently, photovoltaic parameters of the OSCs under different compression rates are evaluated (Figures 6b–e). *I*_sc_ exhibits a continuous decrease as a function of compression rate mainly due to the change of active area, while no significant variation is observed in *V*_oc_ and FF. After the 100‐cycle stretching/compression tests, the ZnO‐based device maintains 82% of the original efficiency and the IZO‐based device maintains 83% (Figure 6f). Due to the combined high efficiency and durable mechanical stability, the ultrathin flexible OSCs fabricated with the IZO ETL show great potential for application in wearable devices.

## 3 Conclusion

In conclusion, a sol–gel processed IZO ETL is developed with improved electronic properties and reduced annealing temperature. Inverted PM6:L8‐BO OSCs based on IZO ETLs exhibit an efficiency of 17.8%, significantly higher than those based on ZnO ETLs (17.0%). The doping of In can effectively improve electron extraction and suppress trap‐assisted charge recombination. In addition, IZO‐based devices exhibit excellent stability, maintaining 95% of their initial efficiency after 2000 h of storage in an N_2_ environment, while ZnO‐based devices only retain 87% of their initial efficiency. Due to its low annealing temperature, IZO ETLs can also be used in flexible OSCs. An ultrathin flexible OSCs is developed based on IZO ETLs, achieving an efficiency of 17.0% and a power‐per‐weight ratio of 40.4 W g^−1^, associated with excellent mechanical stability. These results suggest that IZO is a promising electron transport layer for both high‐efficiency rigid and flexible OSCs, which paves the way for industrial applications.

## Conflict of Interest

The authors declare no conflict of interest.

## Supporting information

Supporting Information

## Data Availability

The data that support the findings of this study are available from the corresponding author upon reasonable request.
