# Supplementary material for: In‐Doped ZnO Electron Transport Layer for High‐Efficiency Ultrathin Flexible Organic Solar Cells
Source: Adv Sci (Weinh). 2024 Jun 25;11(37):2402158. doi: 10.1002/advs.202402158 (PMC11462292; doi:10.1002/advs.202402158)
Supplement: Supplementary file 1 — Supporting Information [file ADVS-11-2402158-s001.docx]

Supporting Information

**In-doped ZnO Electron Transport Layer for High-Efficiency Ultrathin Flexible Organic Solar Cells**

*Xiujun Liu, Yitong Ji, Zezhou Xia, Dongyang Zhang, Yingying Cheng, Xiangda Liu, Xiaojie Ren, Xiaotong Liu, Haoran Huang, Yanqing Zhu, Dr. Xueyuan Yang, Dr. Xiaobin Liao, Prof. Long Ren, Dr. Wenliang Tan, Prof. Zhi Jiang, Prof. Jianfeng Lu, Prof. Christopher McNeill*, Prof. *Wenchao Huang**

X. Liu, Y. Ji, Z. Xia, D. Zhang, Y. Cheng, X. Liu, X. Ren, X. Liu, H. Huang, Dr. X. Yang, Dr. X. Liao, Prof. W. Huang

State Key Laboratory of Advanced Technology for Materials Synthesis and Processing

School of Materials Science and Engineering

Wuhan University of Technology

Wuhan 430070, P.R. China

E-mail: [wenchao.huang@whut.edu.cn](mailto:wenchao.huang@whut.edu.cn)

Y. Zhu, Prof. J. Lu

State Key Laboratory of Silicate Materials for Architectures

Wuhan University of Technology

Wuhan 430070, P.R. China

Prof. L. Ren

International School of Materials Science and Engineering

Wuhan University of Technology

Wuhan 430070, P.R. China

Dr. W. Tan

Australian Synchrotron

Australian Nuclear Science and Technology Organisation (ANSTO)

Clayton, Victoria 3168, Australia

Prof. Z. Jiang

School of Integrated Circuits

Harbin Institute of Technology (Shenzhen)

Shenzhen 518055, China

Prof. C R. McNeill

School of Materials Science and Engineering

Monash University

Clayton, Victoria 3168, Australia

**Experimental details**

**1. Materials**

All chemical reagents were directly used without further purification. Zinc acetate dihydrate (Zn(CH_3_COOH)_2_•2H_2_O, ≥99.0%), 2-methoxyethanol (anhydrous, 99.8%), ethanolamine (≥99.0%), chlorobenzene (anhydrous, 99.8%), 1,8-diiodooctane (98%)， molybdenum(VI) oxide (99.97%) were purchased from Sigma-Aldrich. Chloroform (≥99.0%) was purchased from Yonghua Chemical Co., Ltd. Indium nitrate hydrate (InN_3_O_9_•xH_2_O, 99.999%) was purchased from Aladdin. PTB7-Th, PBDB-T-2F (PM6), Y6, PY-IT, L8-BO, and BTP-eC9 were purchased from Organtec Ltd. PC_71_BM was purchased from Luminescence Technology Corp. Ag pellet (99.999%) was purchased from Zhongnuo Advanced Material (Beijing) Technology Co., Ltd. ITO glass was purchased from Suzhou Sunyang Solar Technology Co.

**2. Preparation of ZnO Layer by Sol-gel method**

The ZnO sol-gel precursor solution was prepared by dissolving 0.439 g of Zinc acetate dihydrate in 10 ml of 2-methoxy ethanol solution at 80 °C for 30 min on a hot-plate, then adding 122 μL of ethanolamine and continuing to stir for 3 h. It was filtered through a 0.22 μm pore size filter head before use.

IZO (5%) sol-gel precursor solution was prepared by dissolving 0.439 g of zinc acetate dihydrate and 0.03 g of indium nitrate hydrate in 10 ml of 2-methoxy ethanol solution. Otherwise, the process was the same as for ZnO.

**3. Device fabrication**

ITO-coated glass substrates were rinsed with detergent, deionized water, ethanol, acetone, and isopropyl alcohol by ultrasonication. The substrates were dried with N_2_ and plasma-cleaned for 5 min. ZnO or IZO(5%) precursor solution was spin-coated on top of ITO substrates at 2000 rpm for 1 min and annealed at 140 ℃ for 20 min. Then, it was immediately transferred to a glove box filled with N_2_. The active layer precursor solutions were prepared as follows: PTB7-Th:PC_71_BM 1:1.5, w/w) (20 mg/mL total, in CB/1,8-diiodoctane (98.5:1.5 v/v)), PM6:PY-IT (1:1, w/w) (14 mg/mL total, in CF/1-chloronaphthalene (99:1 v/v)), PM6:Y6 (1:1.2, w/w) (16 mg/mL total, in CF/1-chloronaphthalene (99.5:0.5 v/v)), PM6:L8-BO (1:1.2, w/w) (14.3 mg/mL total in CF/1,8-diiodoctane (99.75:0.25 v/v)), PM6:L8-BO:BTP-eC9 (1:1:0.2, w/w) (14.3 mg/mL total in CF/1,8-diiodoctane (99.75:0.25 v/v). Subsequently, the PM6:L8-BO/PM6:L8-BO:BTP-eC9 blend was spin-coated at 4000 rpm for 35 s and thermally annealed at 100 ℃ for 5 min. The PM6:Y6/PM6:PY-IT blend was spin-coated at 5000 rpm for 35 s and thermally annealed at 100 ℃ for 5 min. The PTB7-Th:PC_71_BM were spin-coated at 1500 rpm for 1 min.The MoO_3_ layer (10 nm) and Ag electrode (100 nm) were deposited on the top of active layer by thermal evaporation under 2×10^−4^ Pa. Inverted ultrathin flexible organic solar cells are prepared on parylene/ITO substrates, other preparation processes are as same as on rigid devices.

**4. Characterizations**

The absorption and transmission spectra were measured with an ultraviolet spectrometer (Shimadzu UV–1900i). Film thickness was measured by a Dektak XT probe profiler (produced by Bruker). SEM images were obtained by JEOL JSM-7500F. AFM images were measured on an atomic force microscope (Cypher ES). The samples for the GIWAXS measurements were prepared on Si substrates. The 10 keV X-ray beam was used as a lightsource with a grazing angle of 0.13–0.17°. The scattered X-rays were detected using a Dectris Pilatus 2M photon counting detector.

The *J–V* characteristics of the devices were measured with a computer-controlled Keithley 2450 Source Measure Unit under AM 1.5G light source (100 mW cm^-2^ ). The device area of 0.04 cm^2^ is defined using a metal shade. The EQE was obtained using a solar cell spectral response measurement system (Enli Technology Co., Ltd., QE-R). The light intensity is calibrated by using a standard Si solar cell. The XPS and UPS experiments were carried out using a Thermo Scientific Escalab 250Xi spectrometer. All procedures are under the N_2_ atmosphere to prevent contamination of the adsorbed impurities. The measurements were performed in the analysis chamber at a base pressure of 10^-10^ mbar The UPS spectra were first calibrated by gold specimens. Dark-state current and capacitance-voltage are tested by using semiconductor parameter analyzer B1500A (Keysight Technology). Transient photocurrent (TPC) and Transient photovoltage (TPV) measurement were measured using a transient photocurrent and photovoltage measurement system (LST-TPC, Shanghai Jinzhu Technology Co., Ltd.).

**
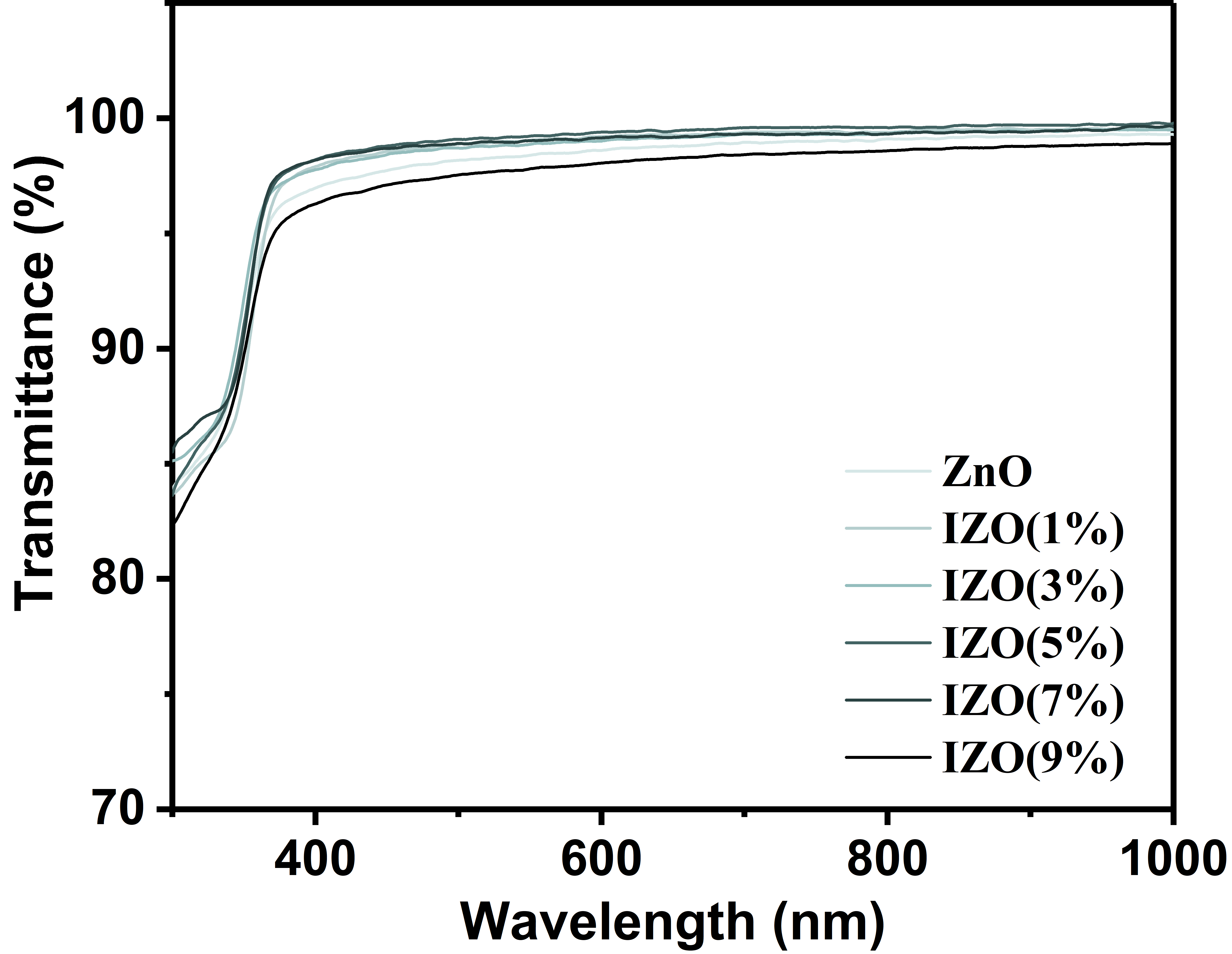
**

**Figure S1.** Transmission spectra of In-doped ZnO films with different contents.

**
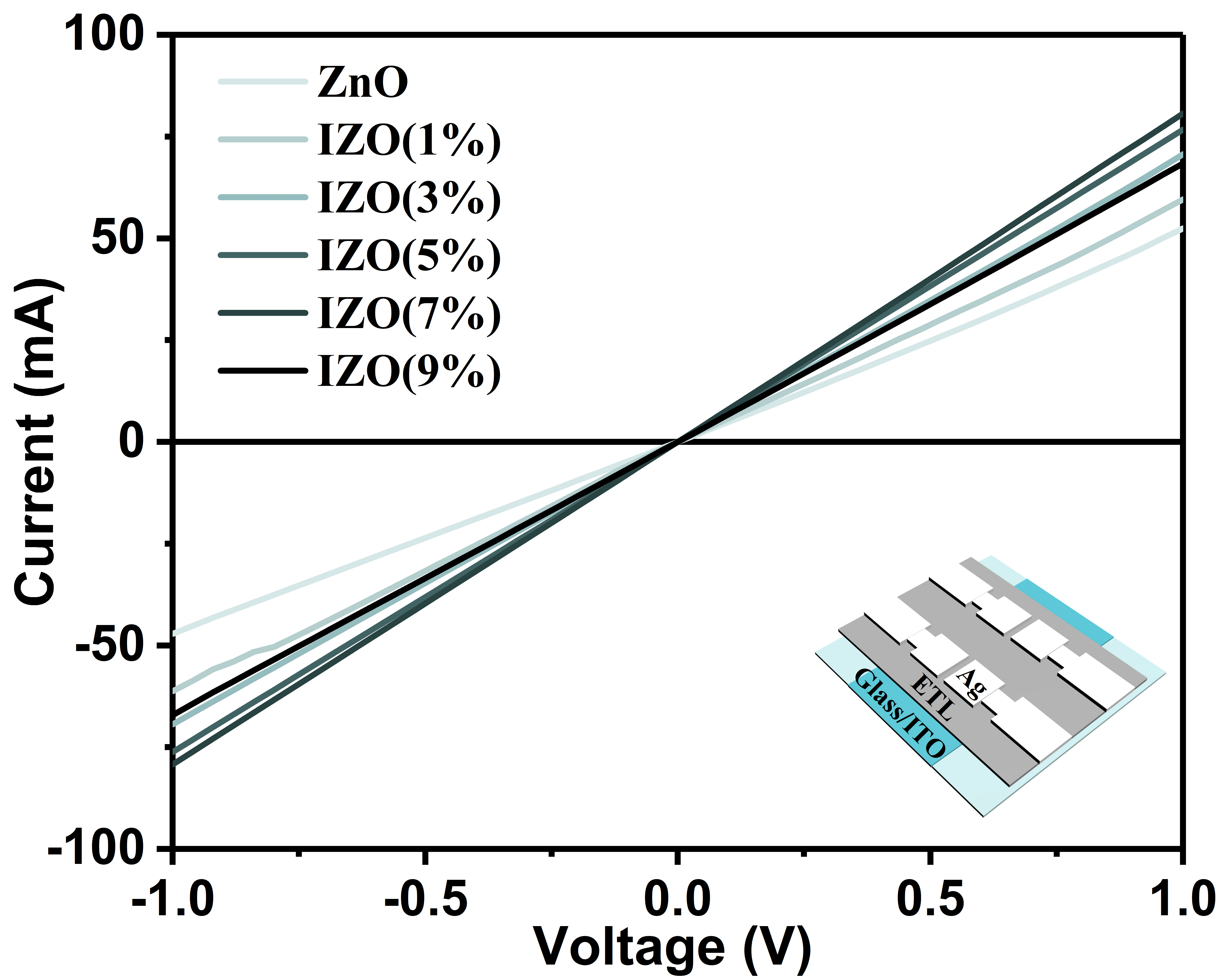
**

**Figure S2.** Comparison of electrical conductivity of ZnO electron transport layer with different contents.


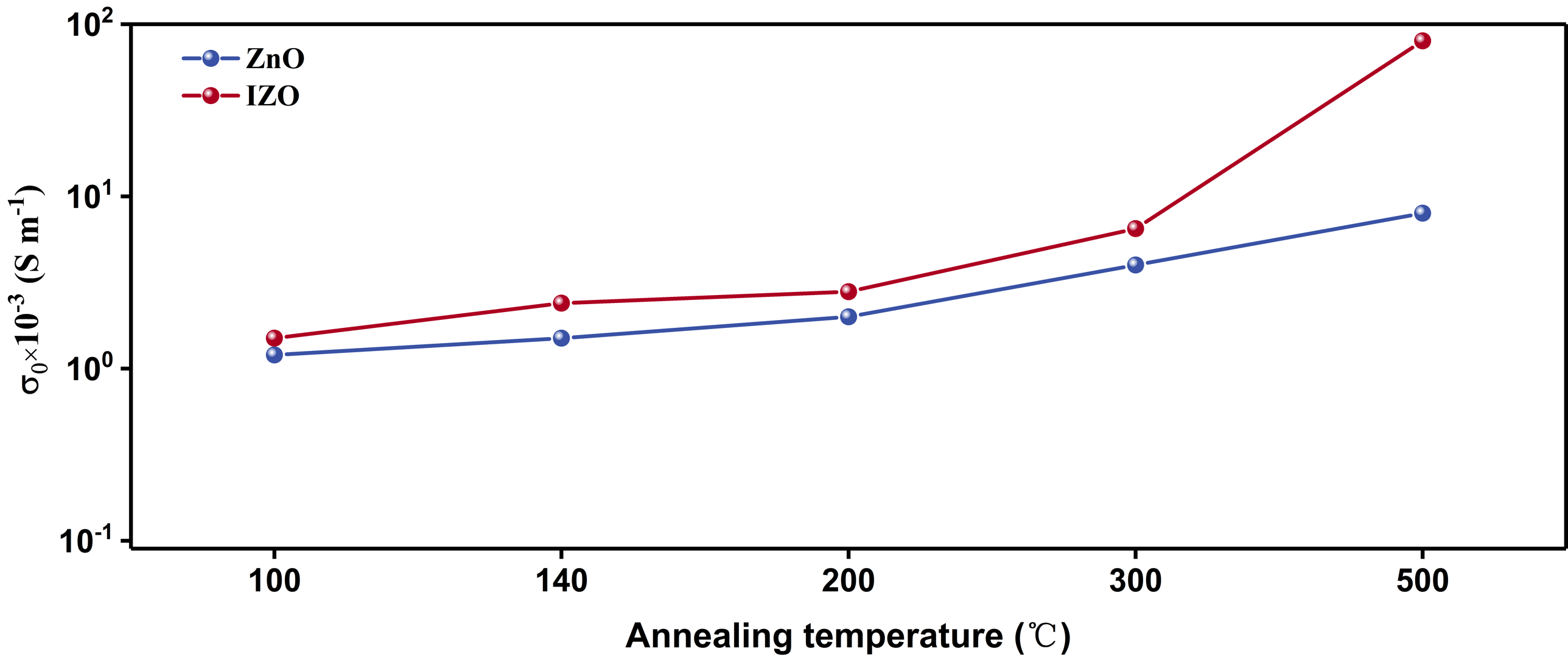


**Figure S3.** The conductivity of pristine ZnO and In-doped IZO (5 mol%) films as a function of annealing temperature.

**
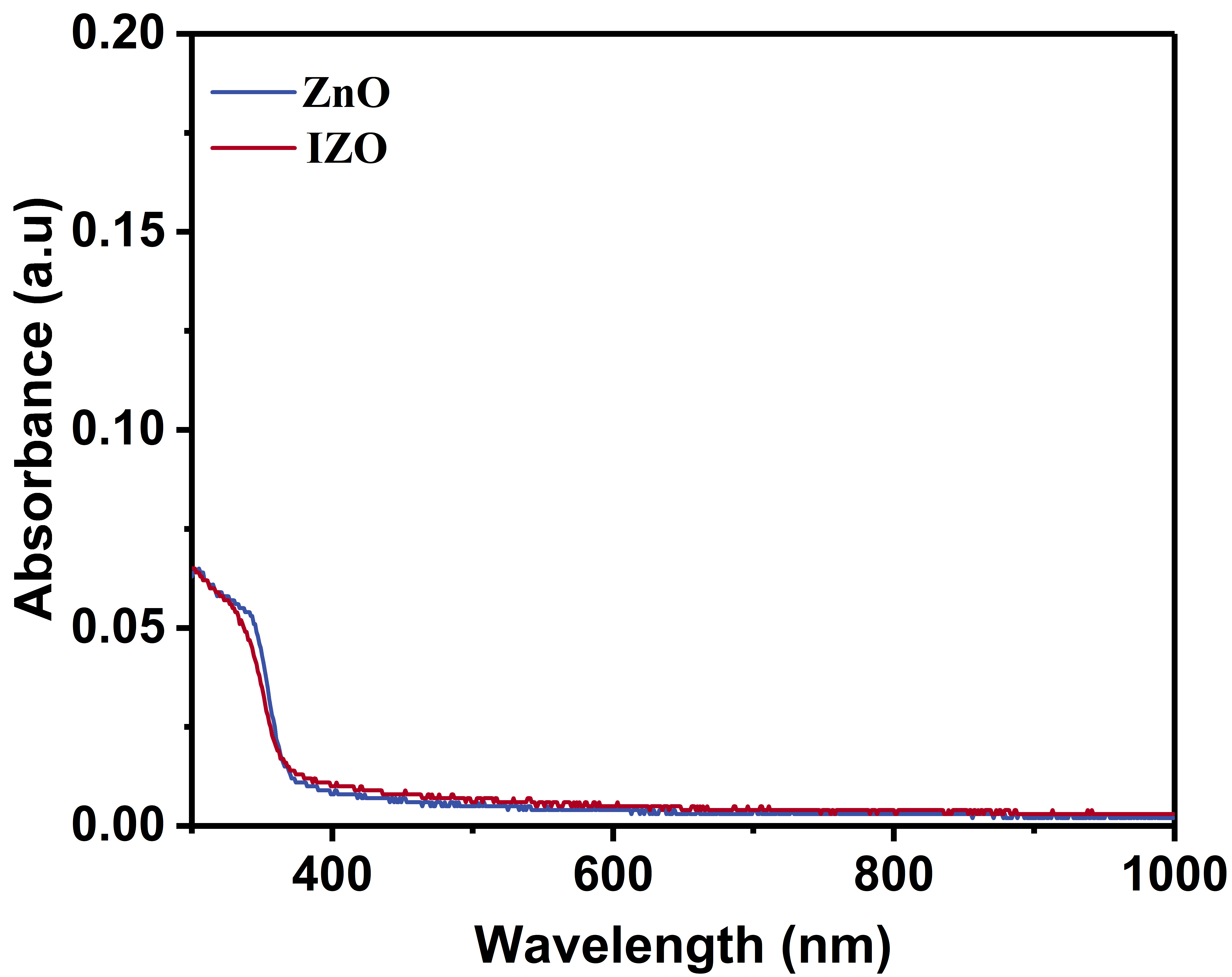
**

**Figure S4.** UV-Vis absorption spectra of ZnO and IZO films.

**
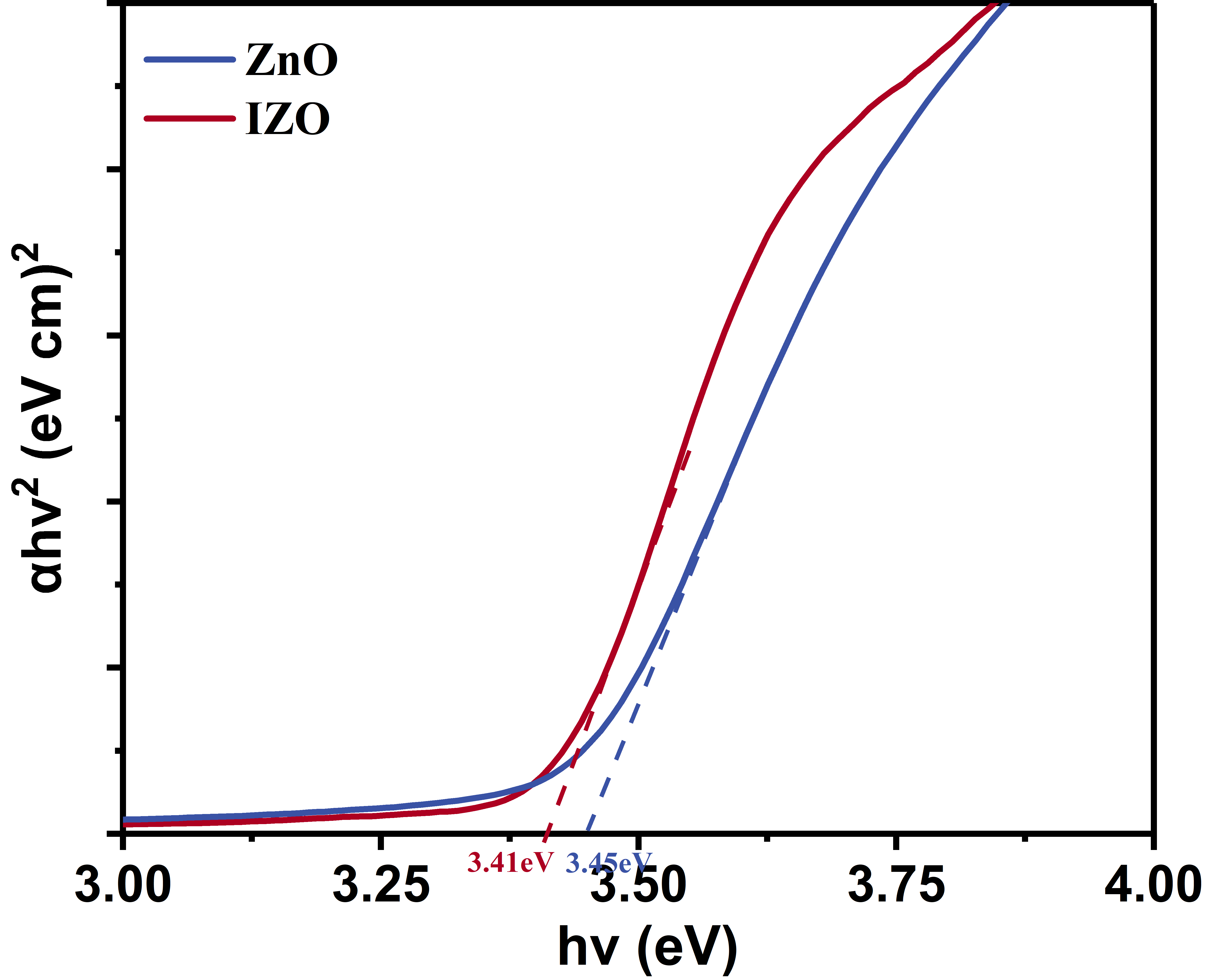
**

**Figure S5** The optical bandgap of the ZnO and IZO calculated from *(αhν)^2^* versus *hν* graph.

**
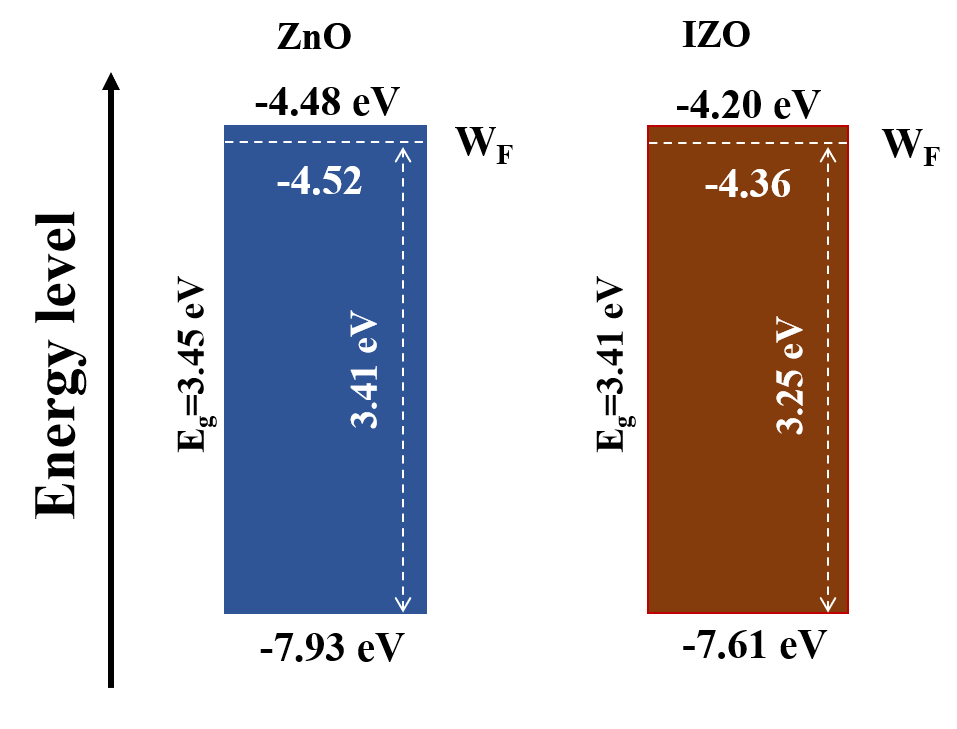
**

**Figure S6.** Energy level diagram of ZnO and IZO films.


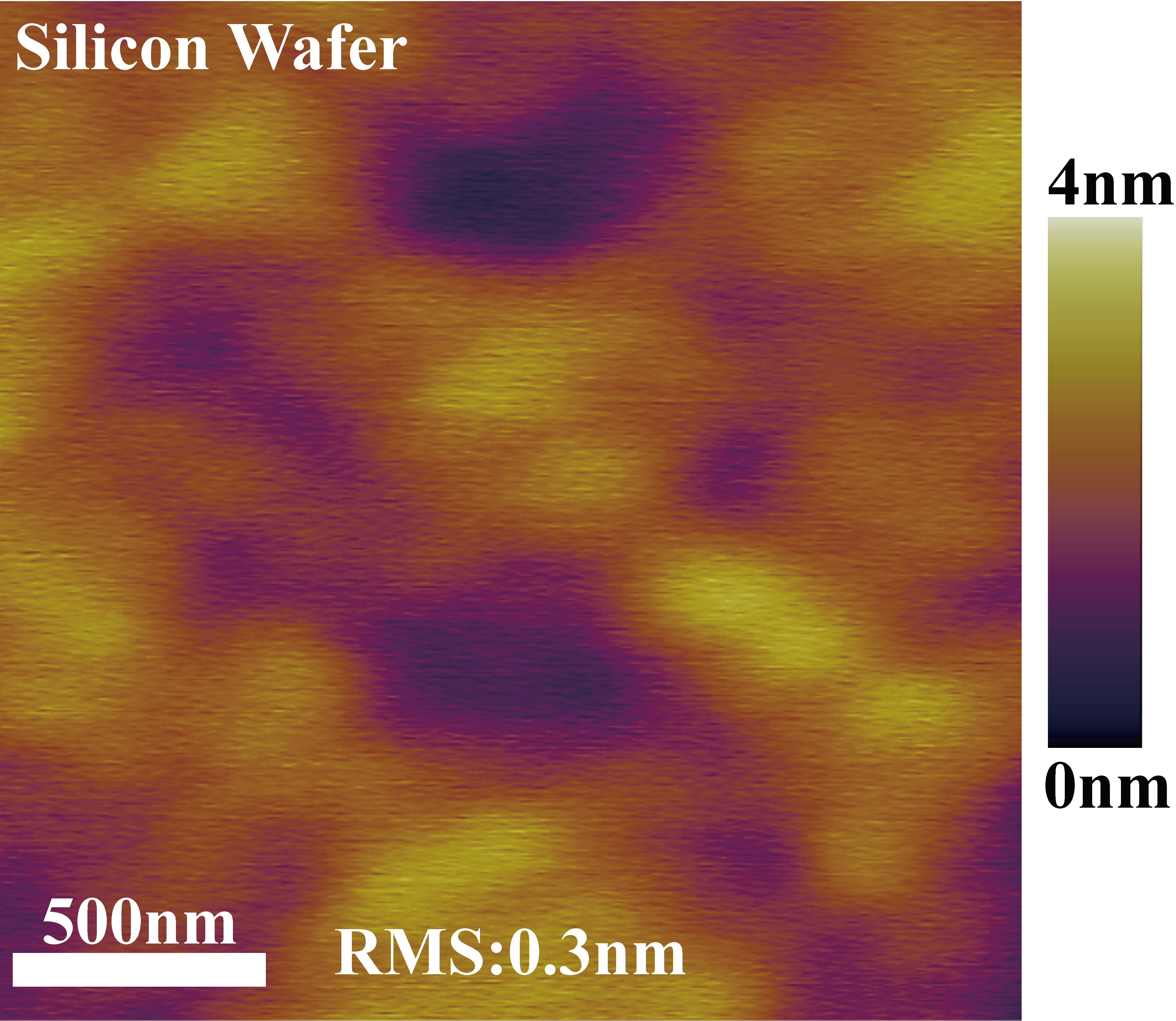


**Figure S7.** AFM images of silicon wafer substrate.

**
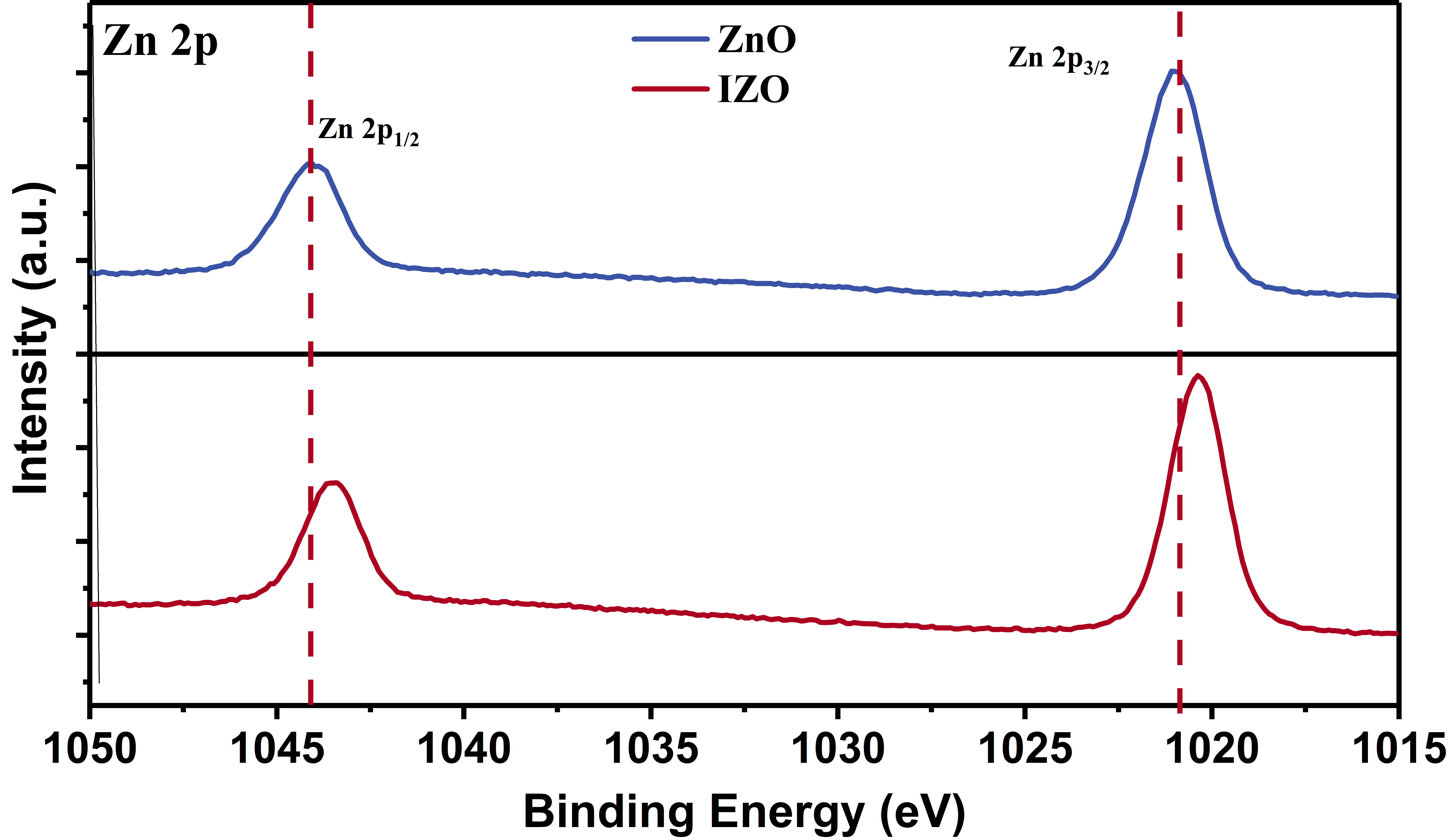
**

**Figure S8.** XPS spectra of Zn 2p in ZnO and IZO thin films.


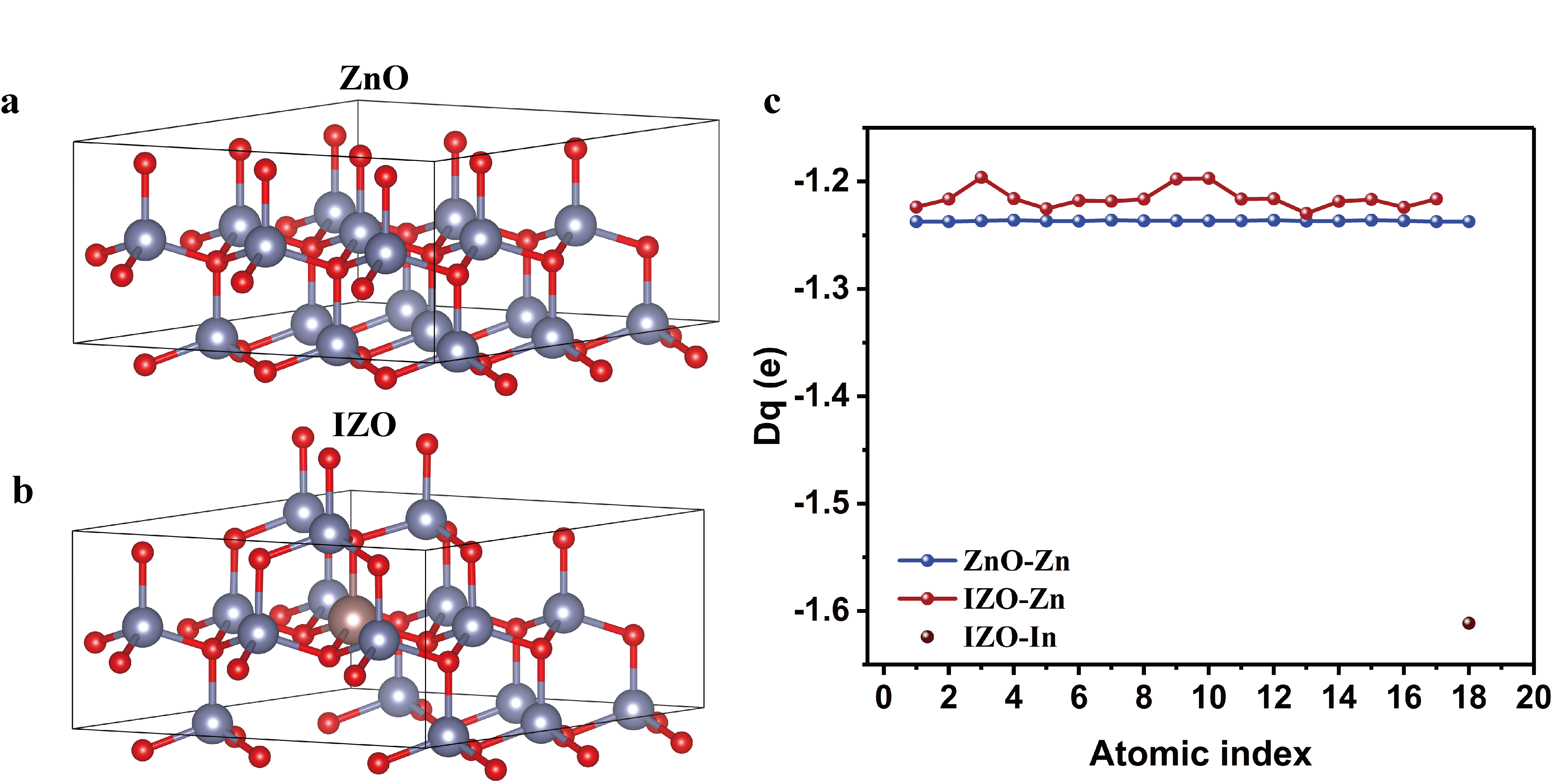


**Figure S9.** Bader charge analysis calculations of ZnO and IZO crystal structures were carried out using DFT theory.


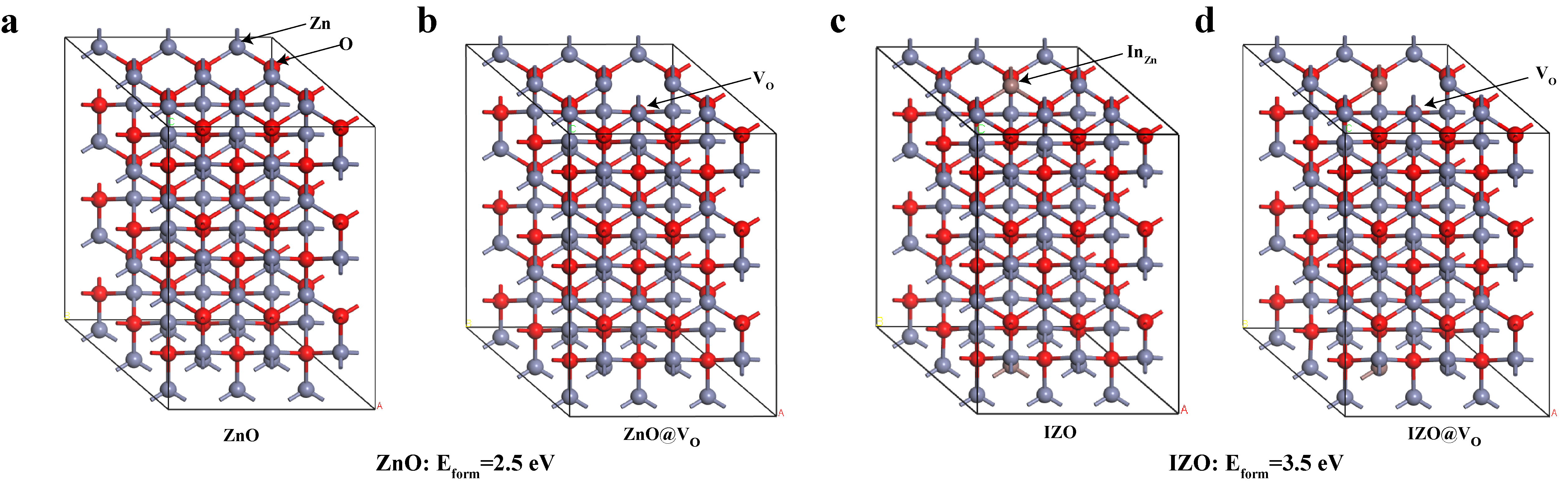


**Figure S10.** The oxygen vacancy formation energy in ZnO and IZO.

**
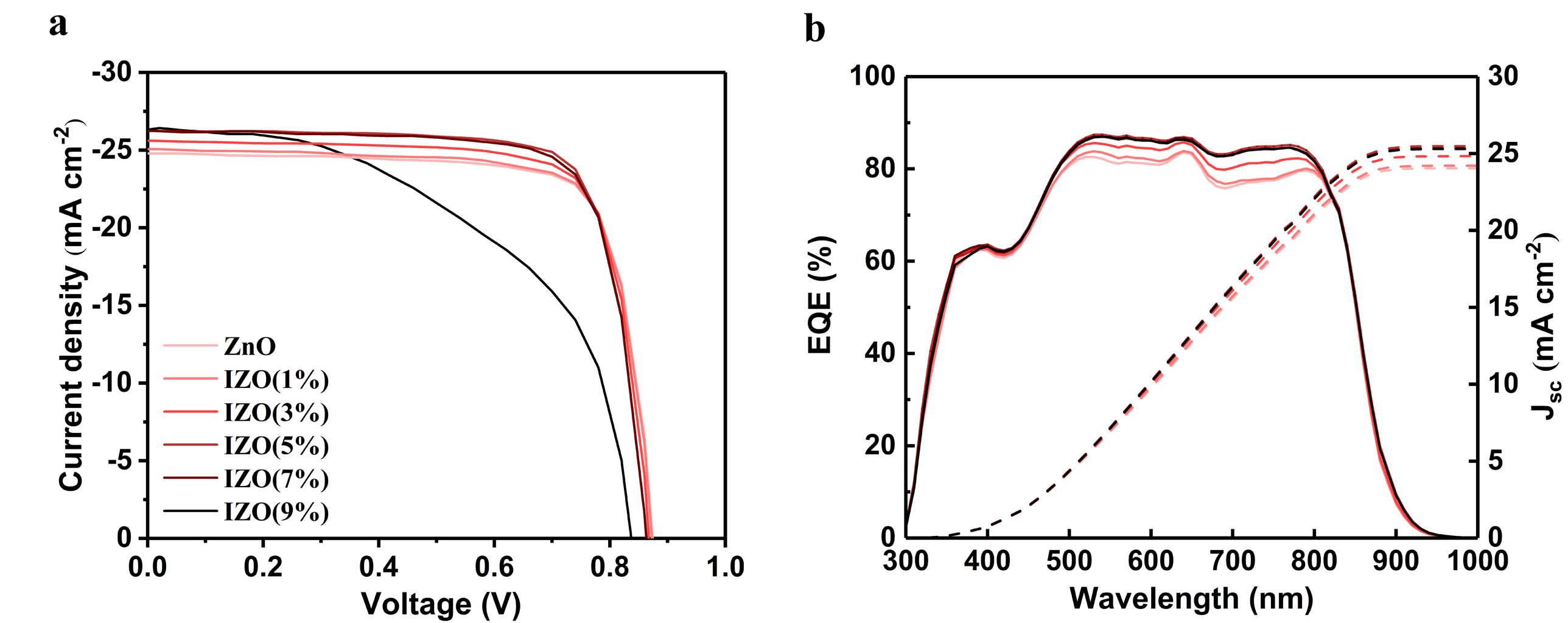
**

**Figure S11.** Device performance of PM6:L8-BO organic solar cells with different contents. (a) *J-V* curve and (b) EQE spectra.

**
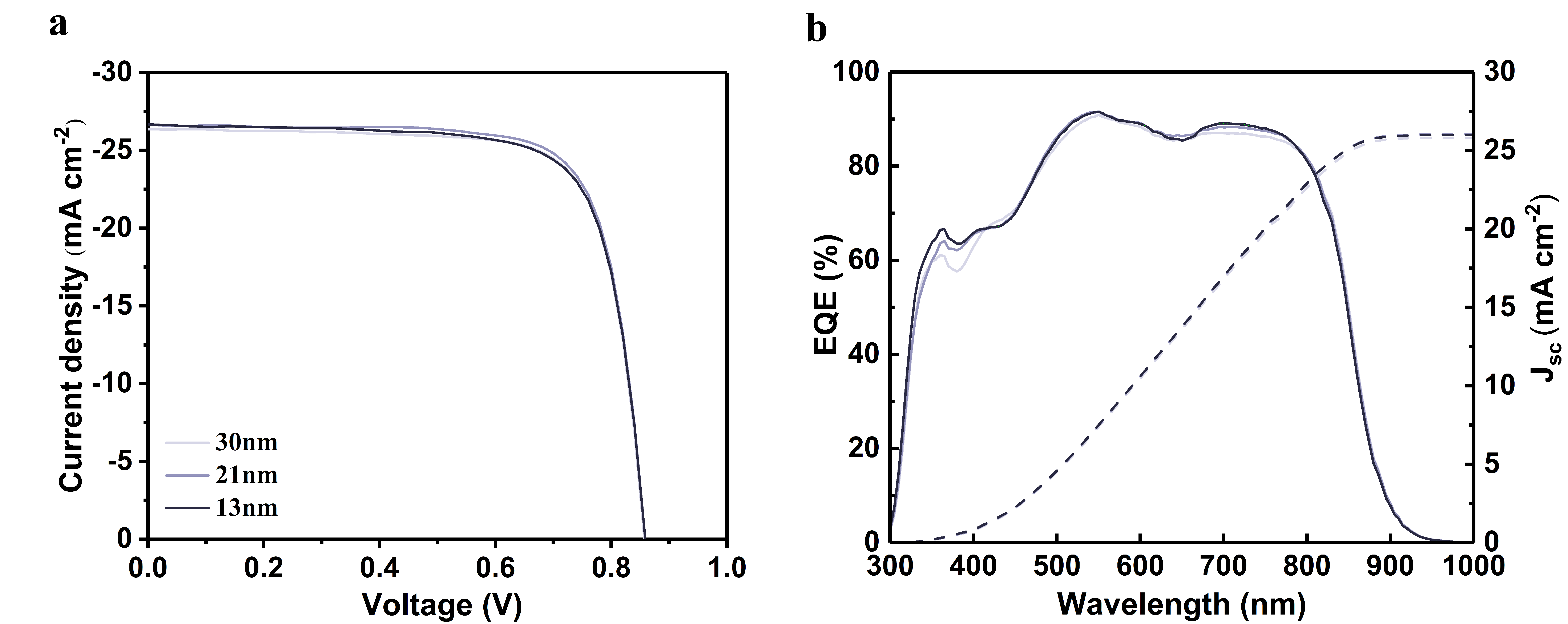
**

**Figure S12.** Device performance of PM6:L8-BO organic solar cells with different IZO thicknesses. (a) *J-V* curve and (b) EQE spectra.


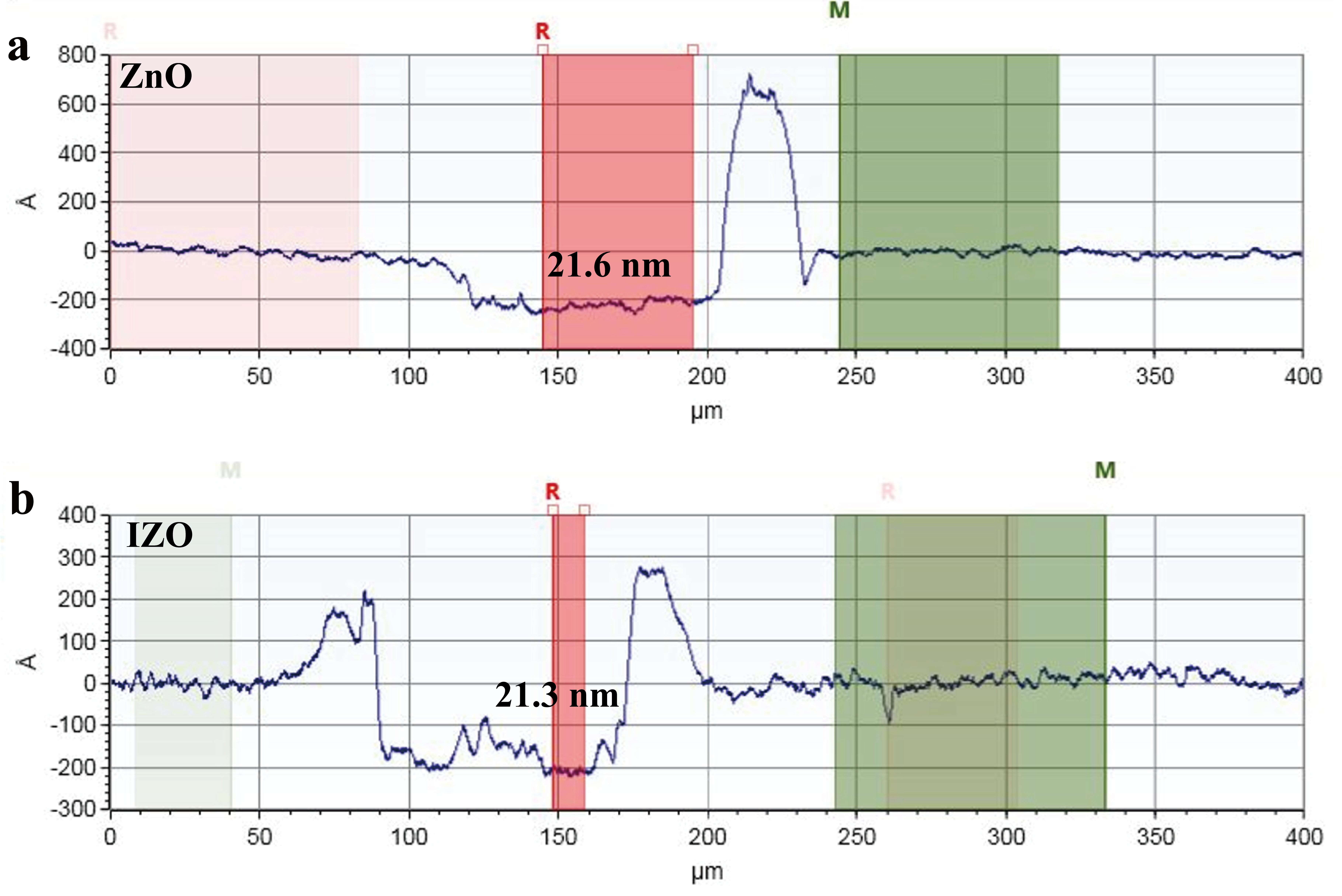


**Figure S13.** Thickness measurements of ZnO and IZO films.

**
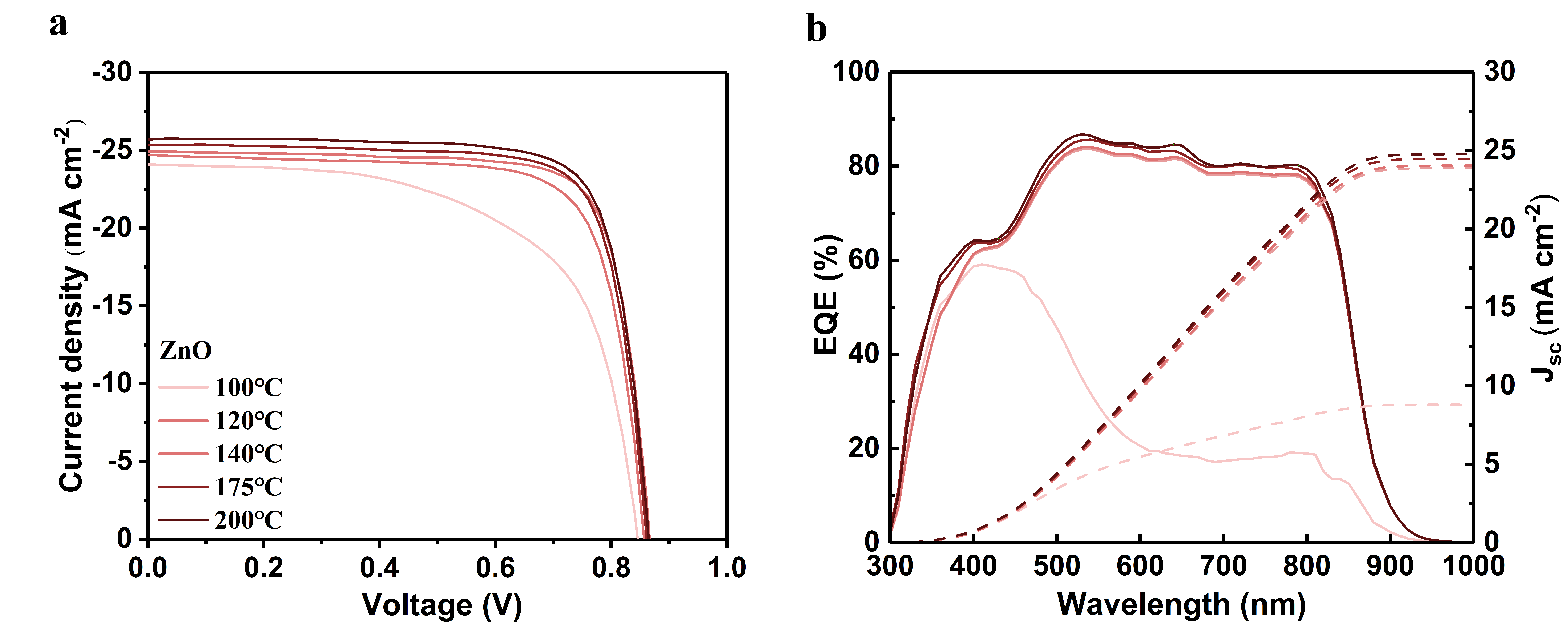
**

**Figure S14.** Device performance of PM6:L8-BO organic solar cells based on ZnO electron transport layer with different annealing temperatures. (a) *J-V* curve and (b) EQE spectra.


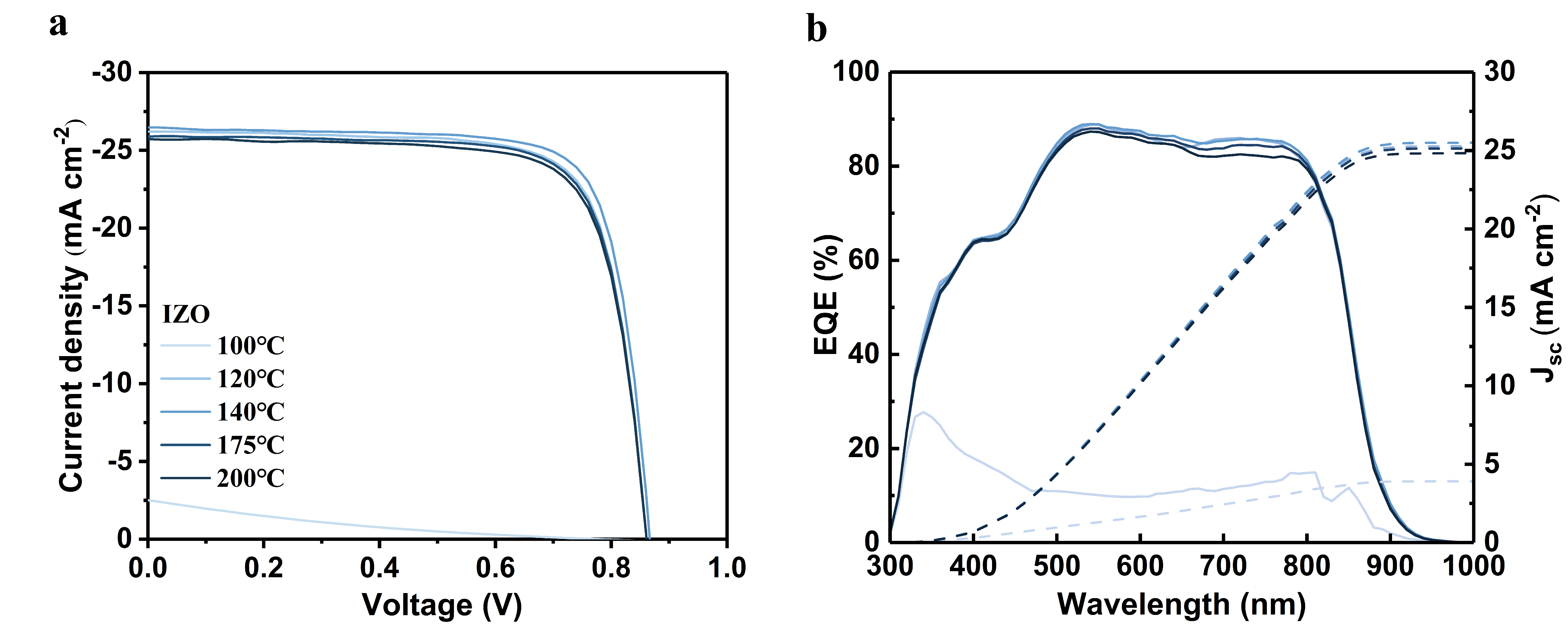


**Figure S15.** Device performance of PM6:L8-BO organic solar cells based on IZO electron transport layer with different annealing temperatures. (a) *J-V* curve and (b) EQE spectra.


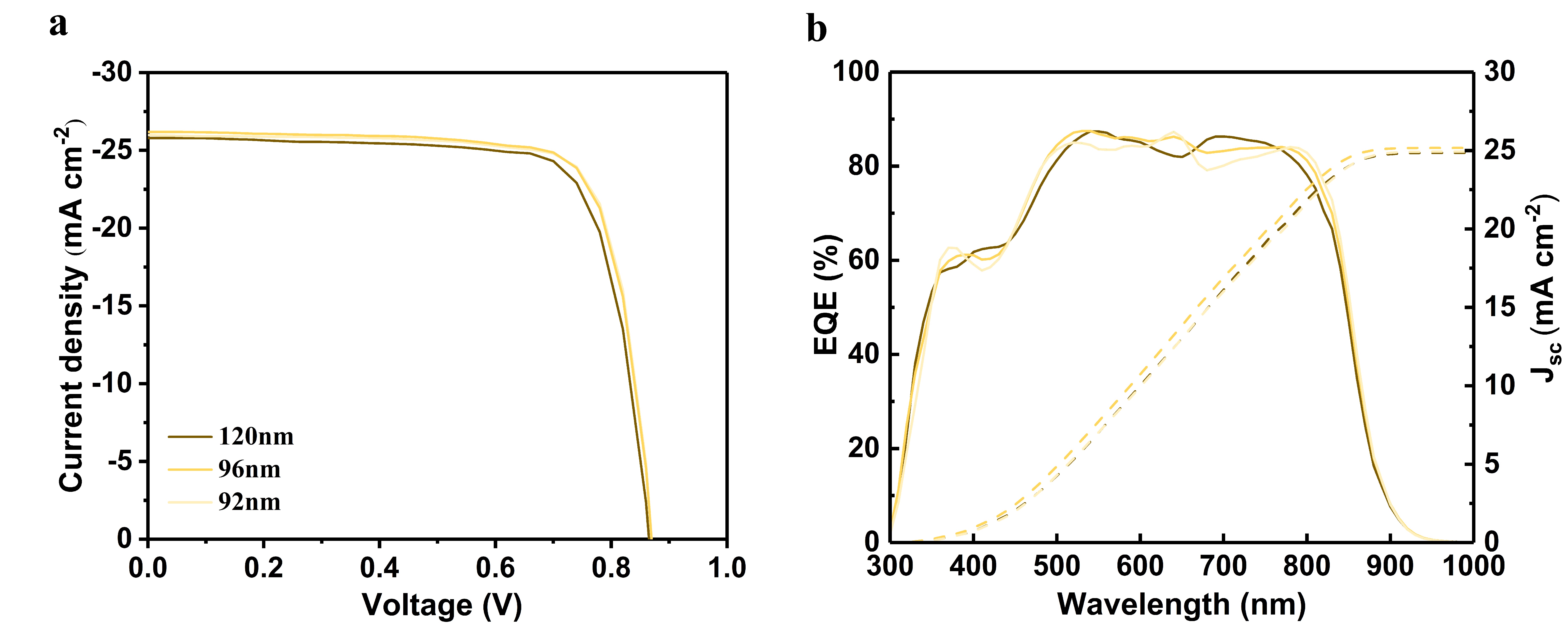


**Figure S16.** Device performance of PM6:L8-BO organic solar cells based on IZO electron transport layer with different active layer thicknesses. (a) *J-V* curve and (b) EQE spectra.

**
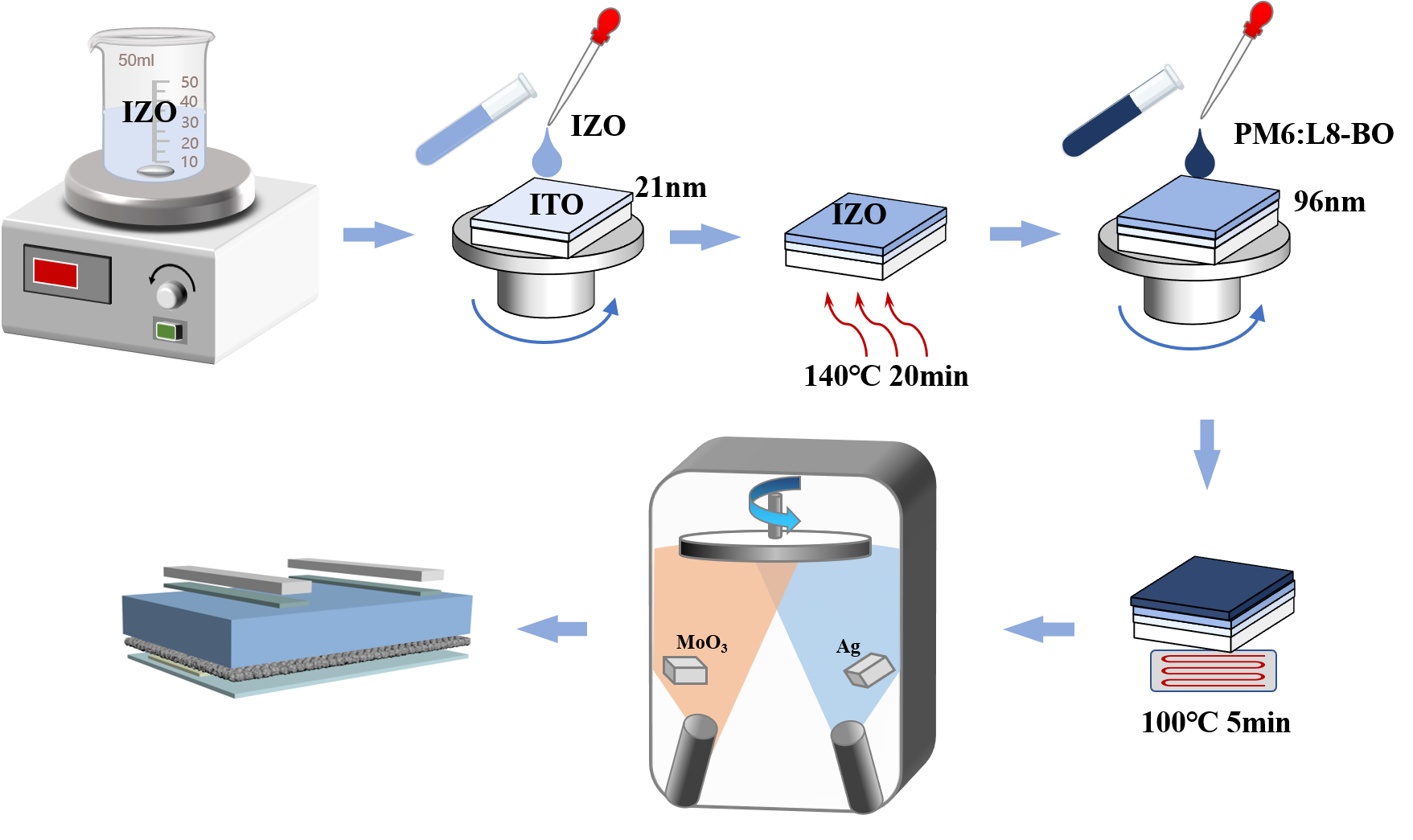
**

**Figure S17.** Scheme of device fabrication.


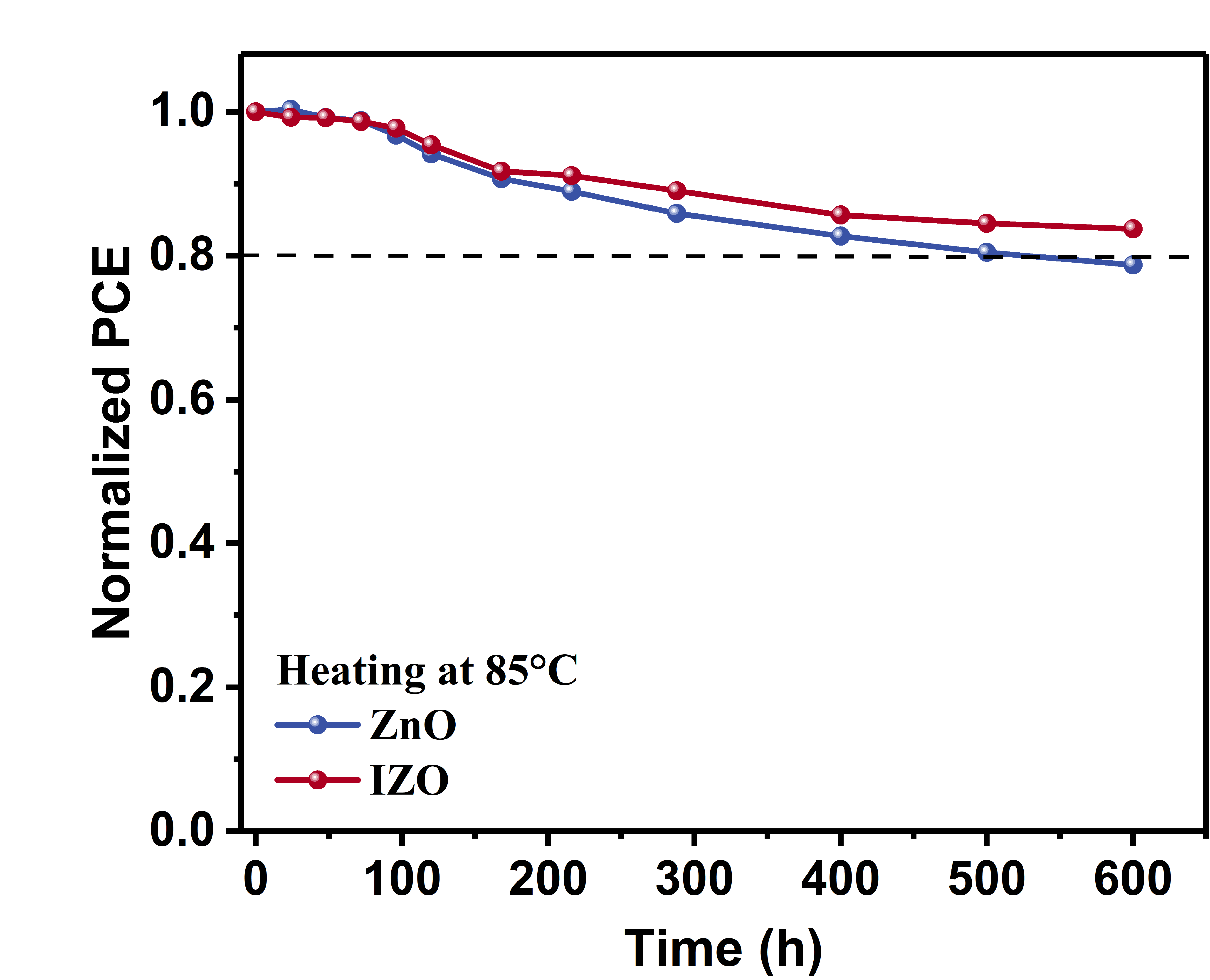


**Figure S18.** Device stability of organic solar cells annealed at 85°C.


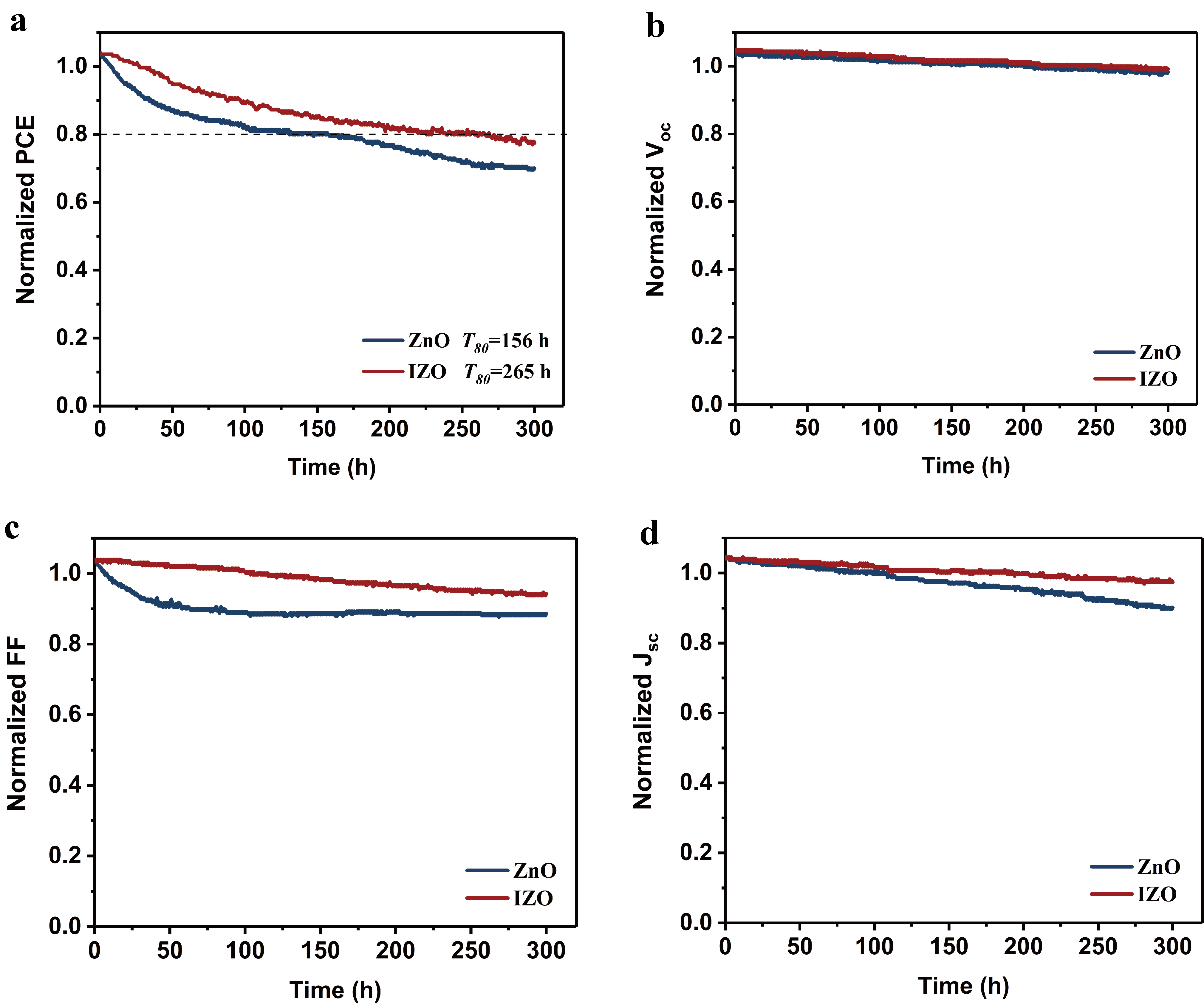


**Figure S19** Normalized (a) PCE, (b) *V_oc_*, (c) FF and (d) *J_sc_* of encapsulated PM6:L8-BO devices under continuous LED light illumination.


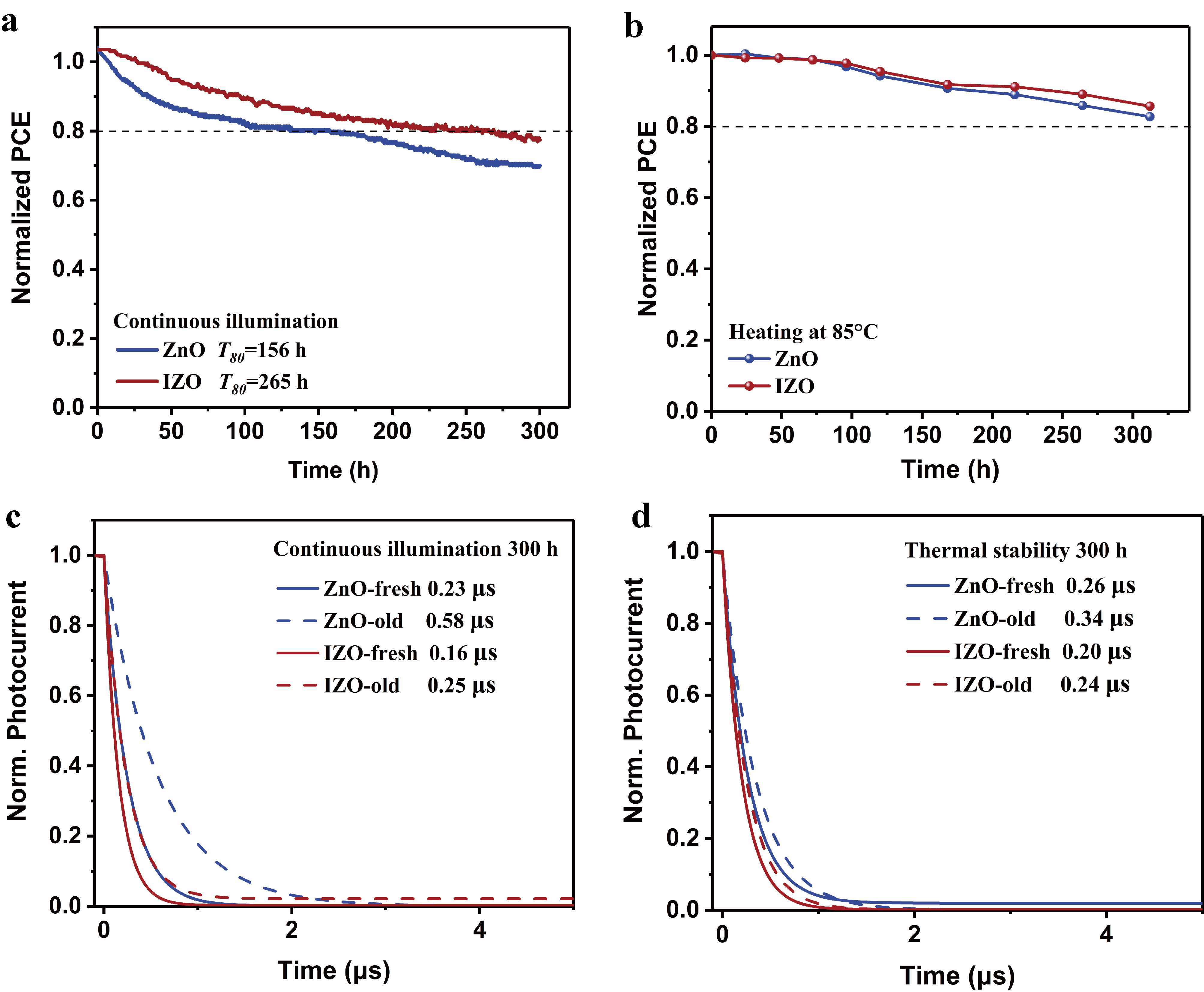


**Figure S20.** Stability test of ZnO and IZO-based organic solar cells under (a) continuous light illumination and (b) thermal-annealing. Comparison of transient photocurrent (TPC) performance of ZnO and IZO-based devices before and after stability tests for (c) light stability and (d) thermal stability.


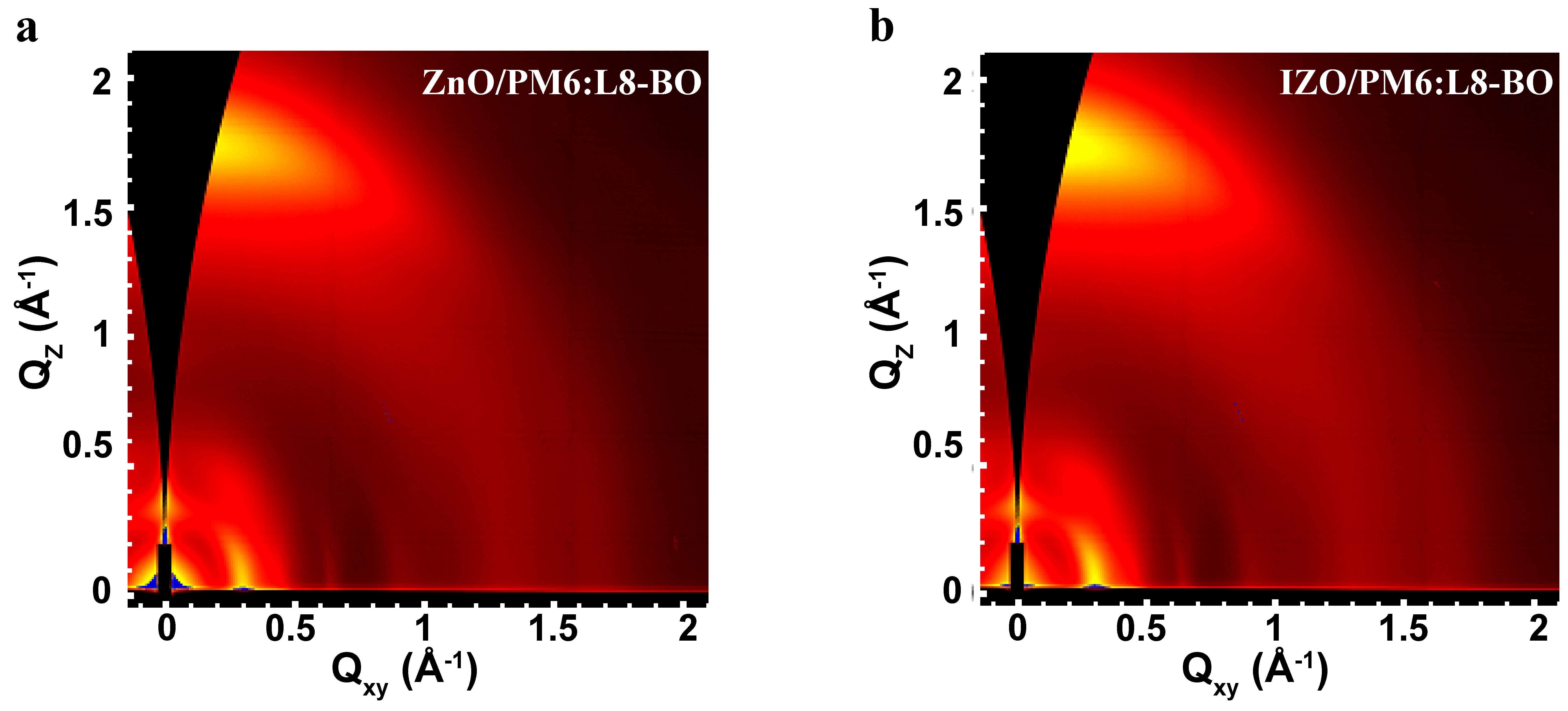


**Figure S21.** 2D GIWAXS scattering patterns of the PM6:L8-BO blend on (a) ZnO ETL and (b) IZO ETL.


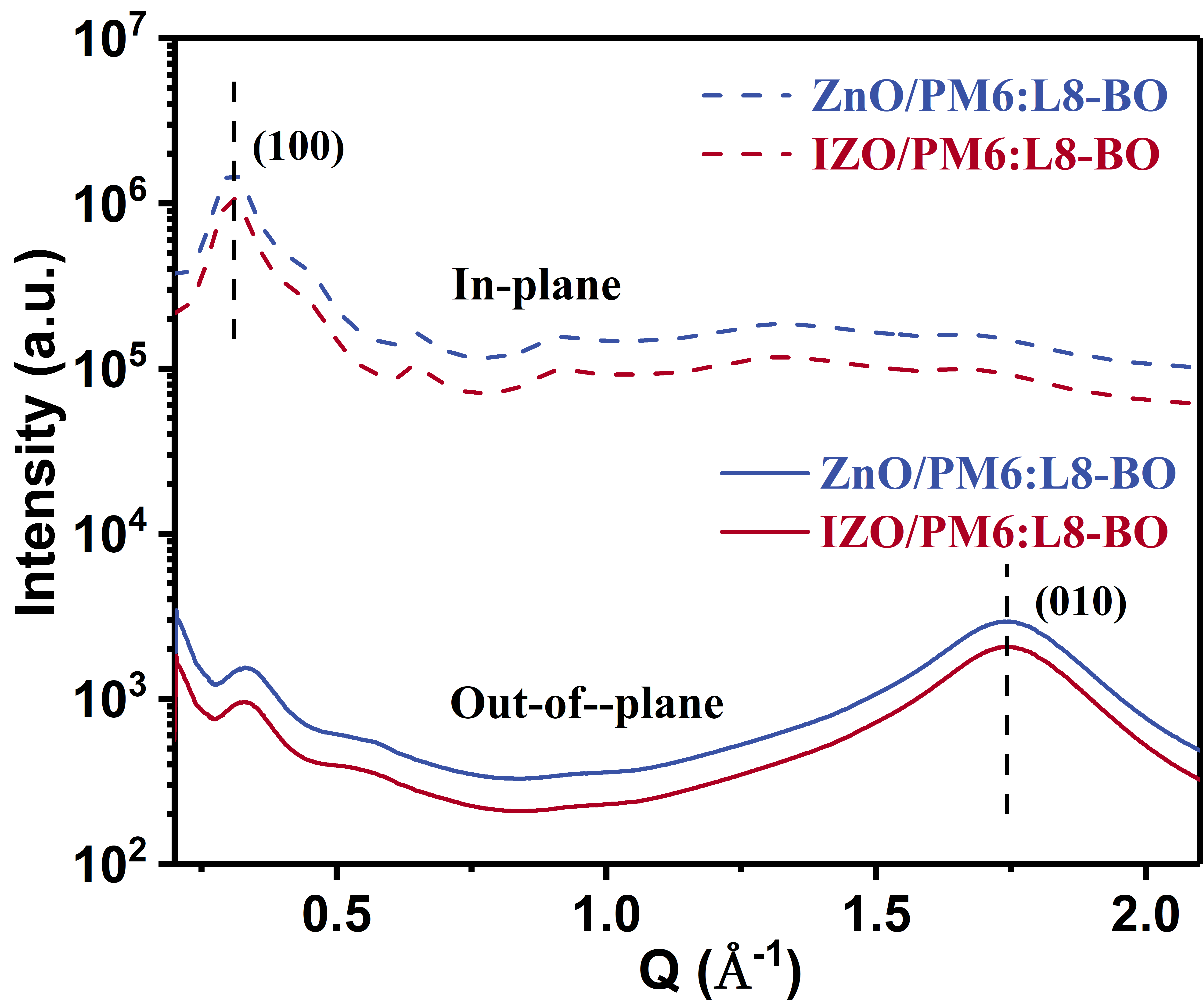


**Figure S22.** Line scattering profiles of PM6:L8-BO blends based on different ELTs cut from 2D GIWAXS patterns along out-of-plane (OOP) direction and in-plane (IP) direction.

**
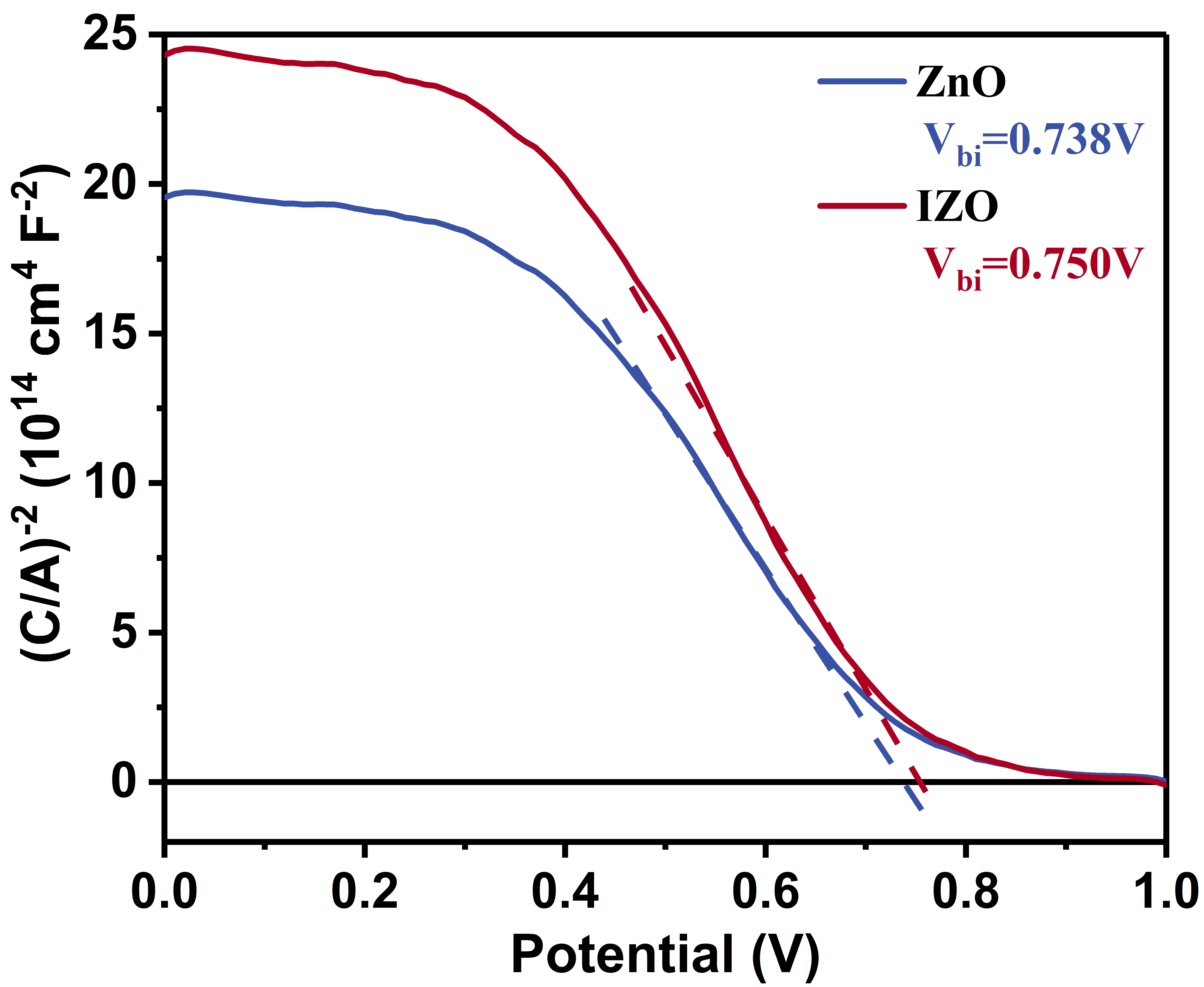
**

**Figure S23.** Mott-Schottky fitting Capacitance-potential diagram of ZnO- and IZO-based devices.

**
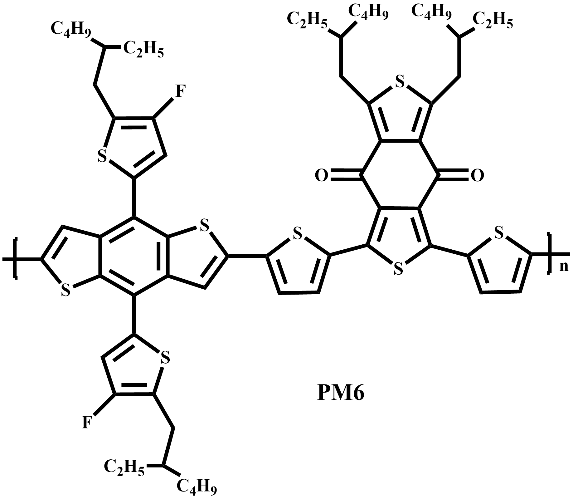

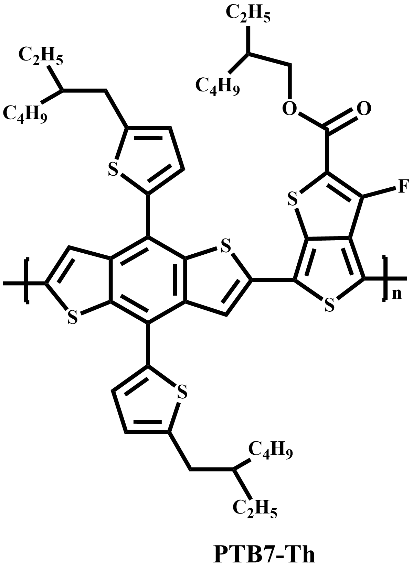
**

**
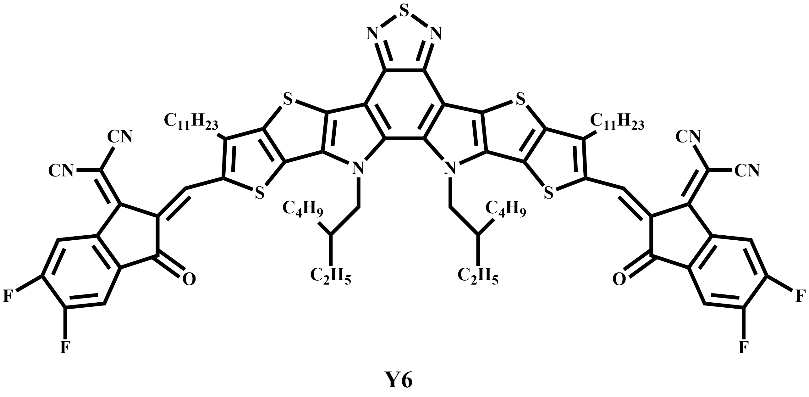

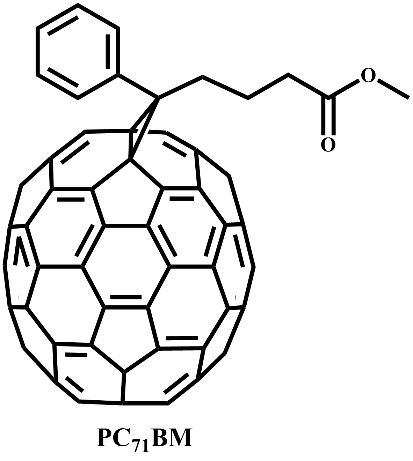
**

**
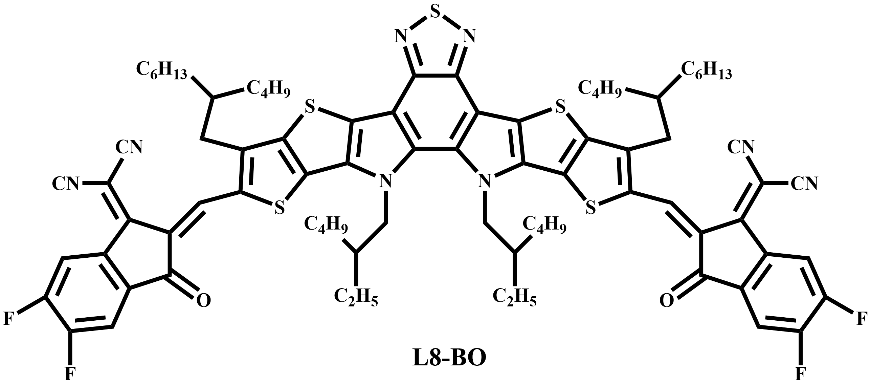
**

**
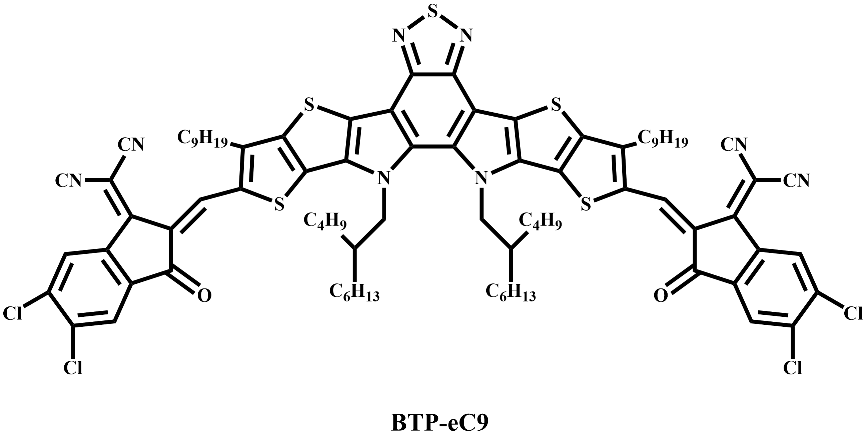
**

**
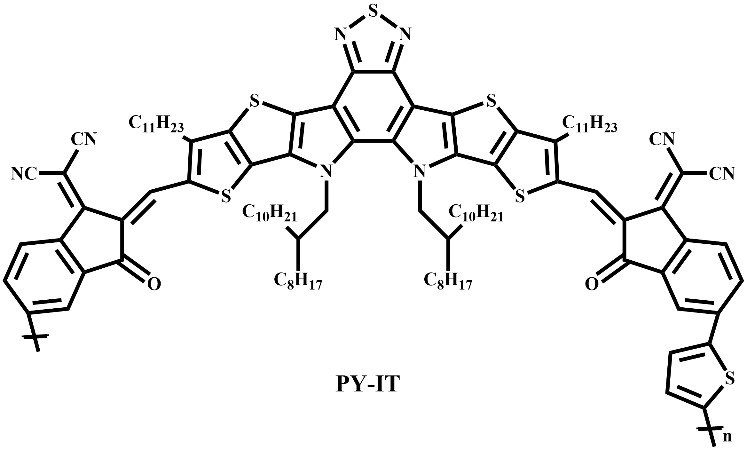
**

**Figure S24.** Chemical Structures of donor and acceptor materials.

**
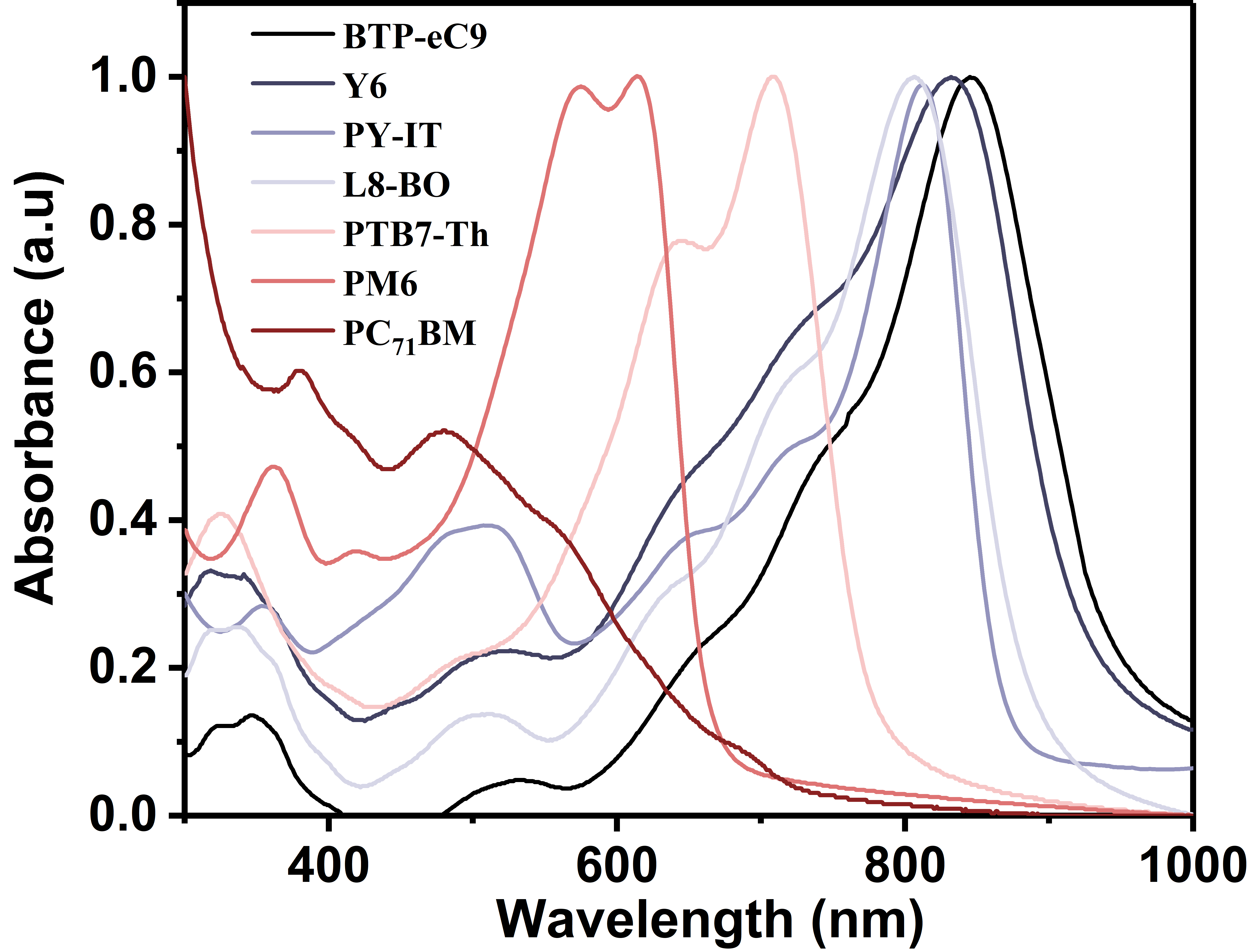
**

**Figure S25.** Absorption spectra of different donor and acceptor materials.


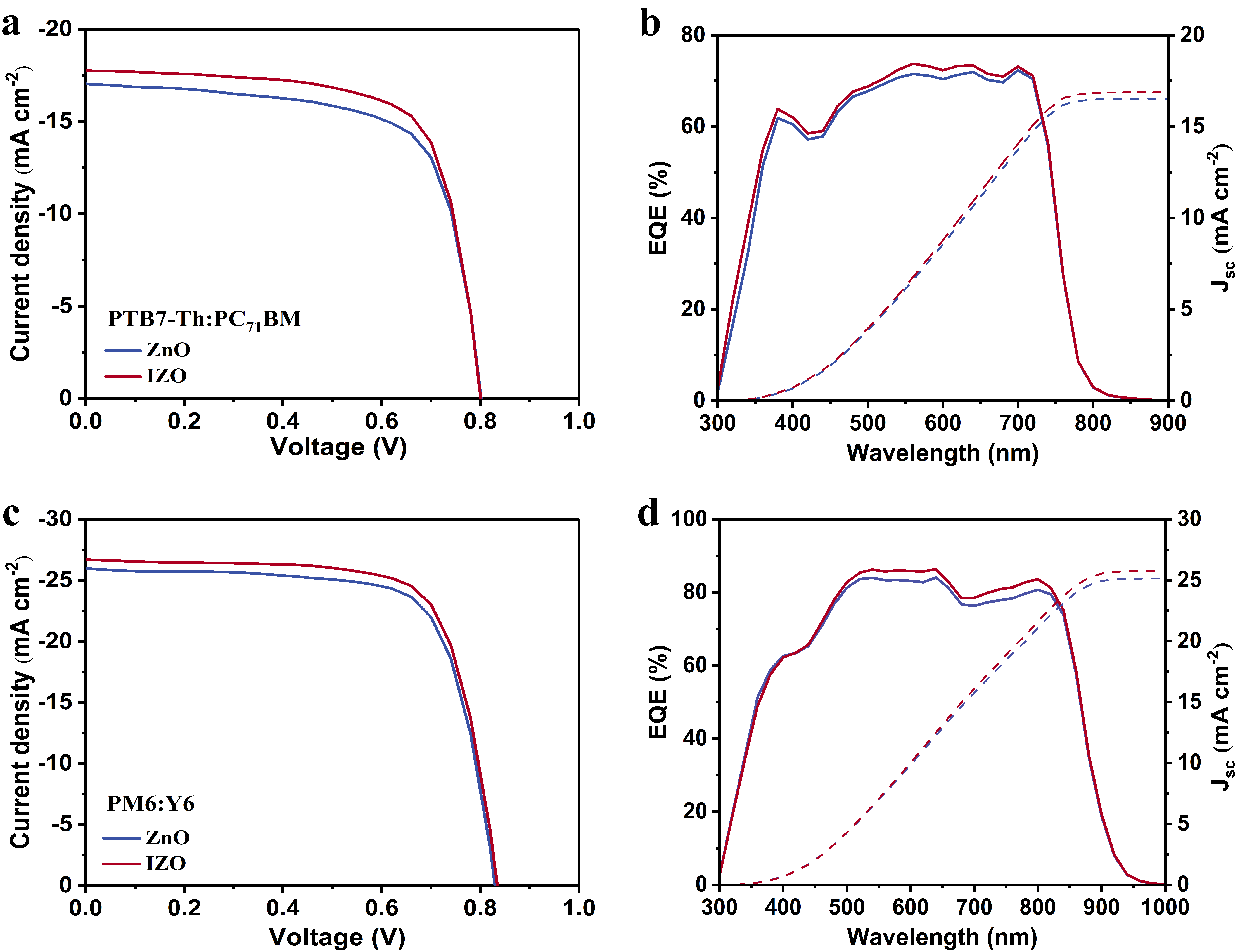


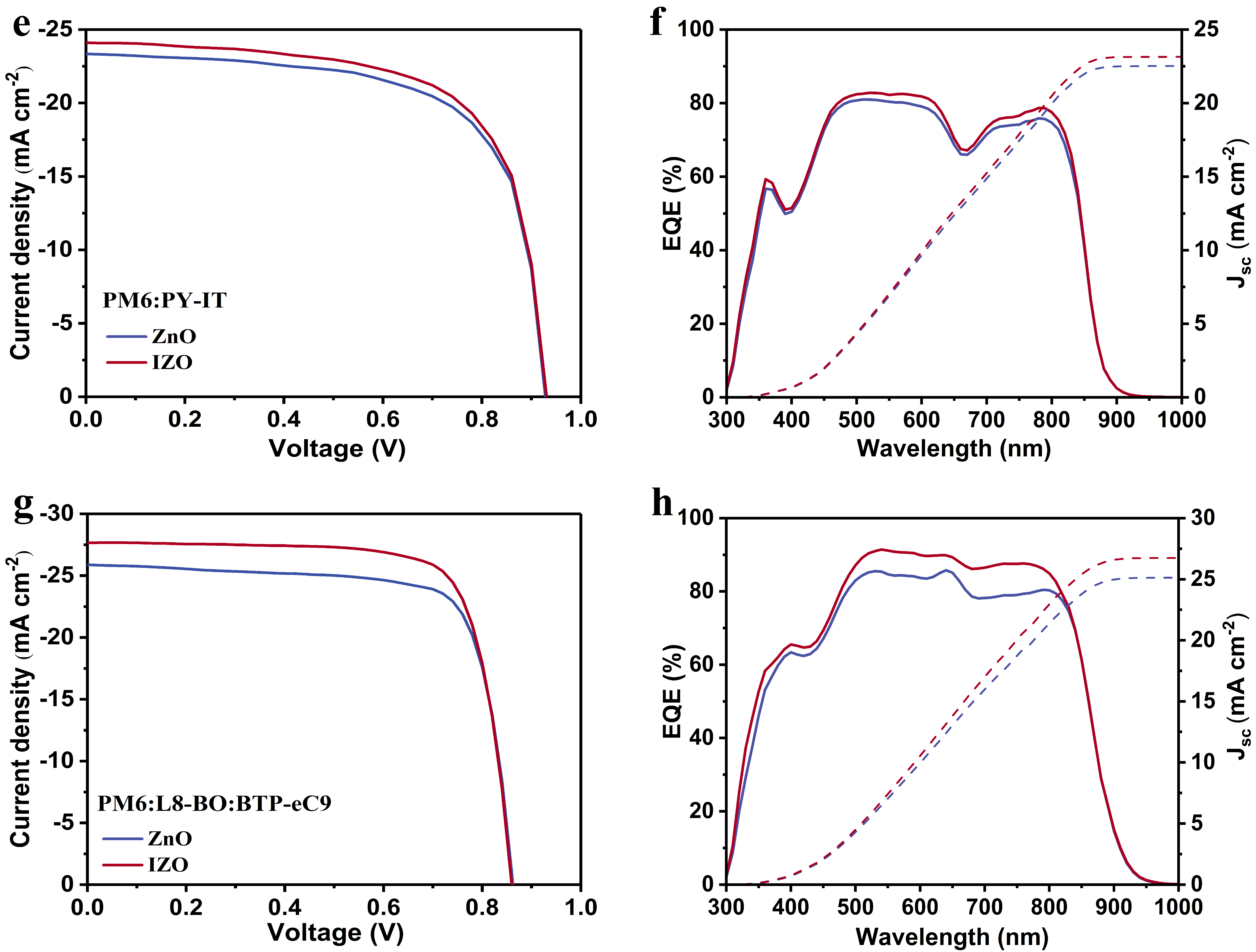


**Figure S26**. *J-V* curves and EQE spectra of inverted organic solar cells. (a, b) PTB7-Th:PC_71_BM, (c, d) PM6:Y6, (e, f) PM6:PY-IT and (g, h) PM6:L8-BO:BTP-eC9.

**
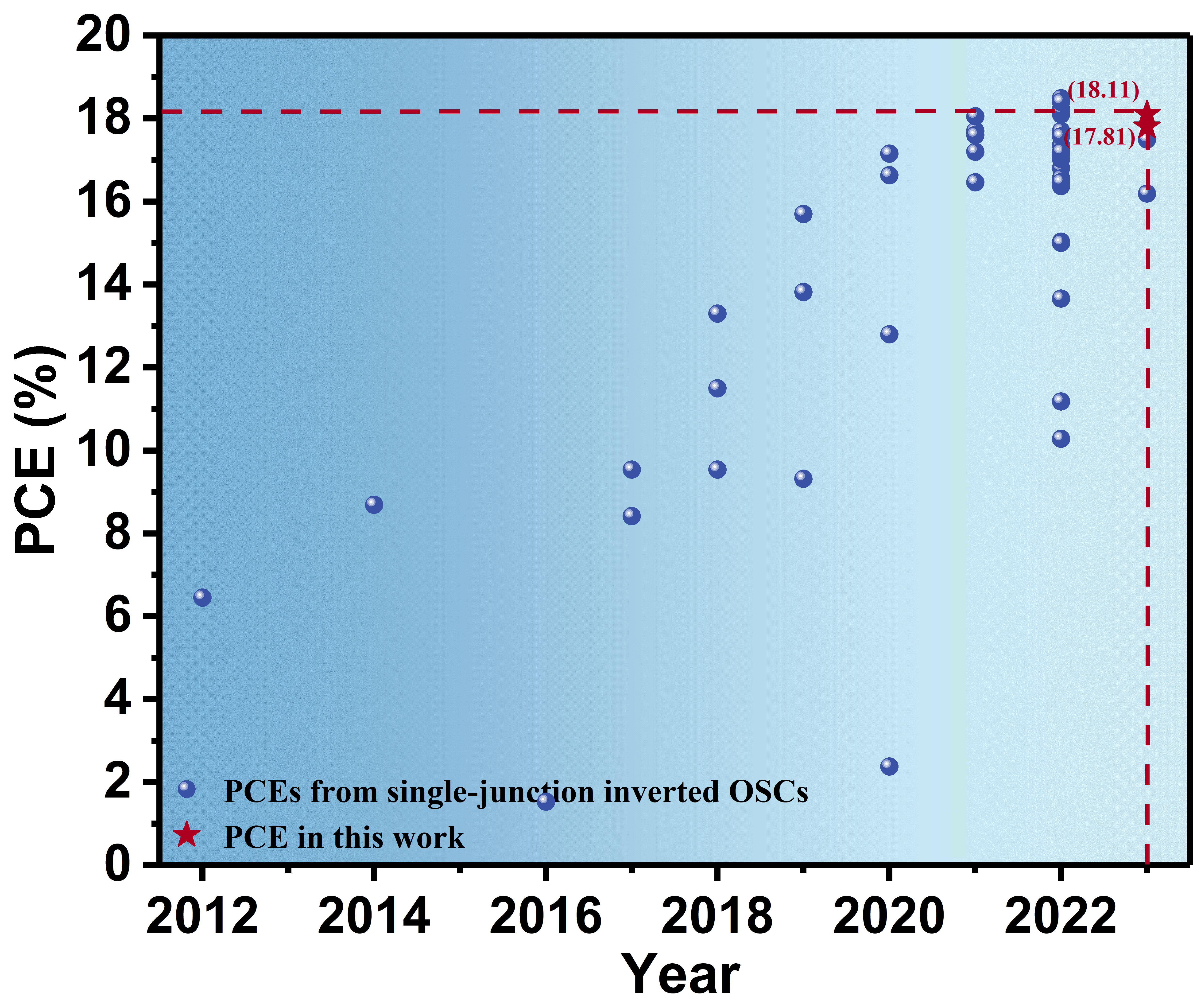
**

**Figure S27.** The summary of the efficiency of inverted rigid orgnaic solar cells.


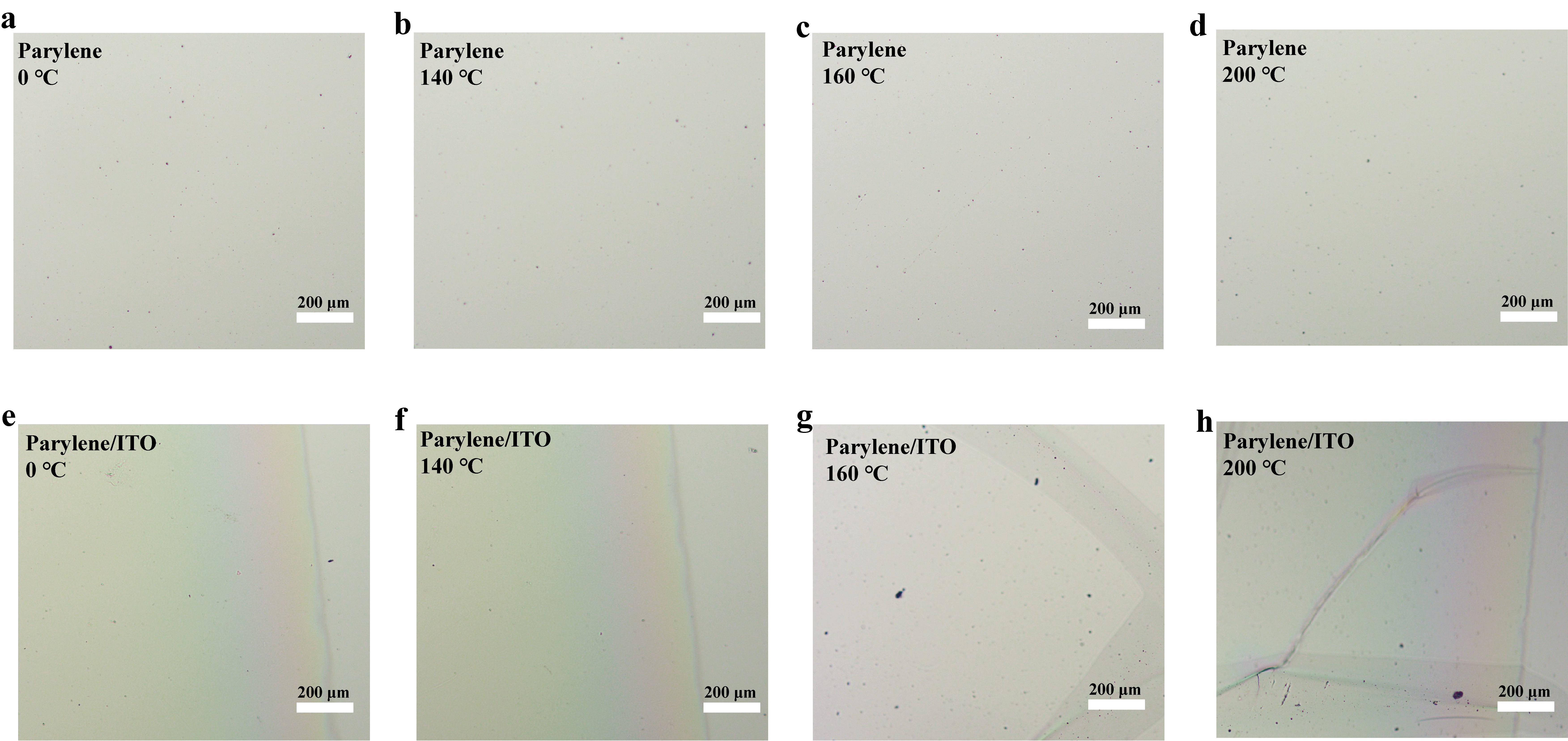


**Figure S28.** Optical images of parylene and parylene/ITO films annealed at different temperatures.

**
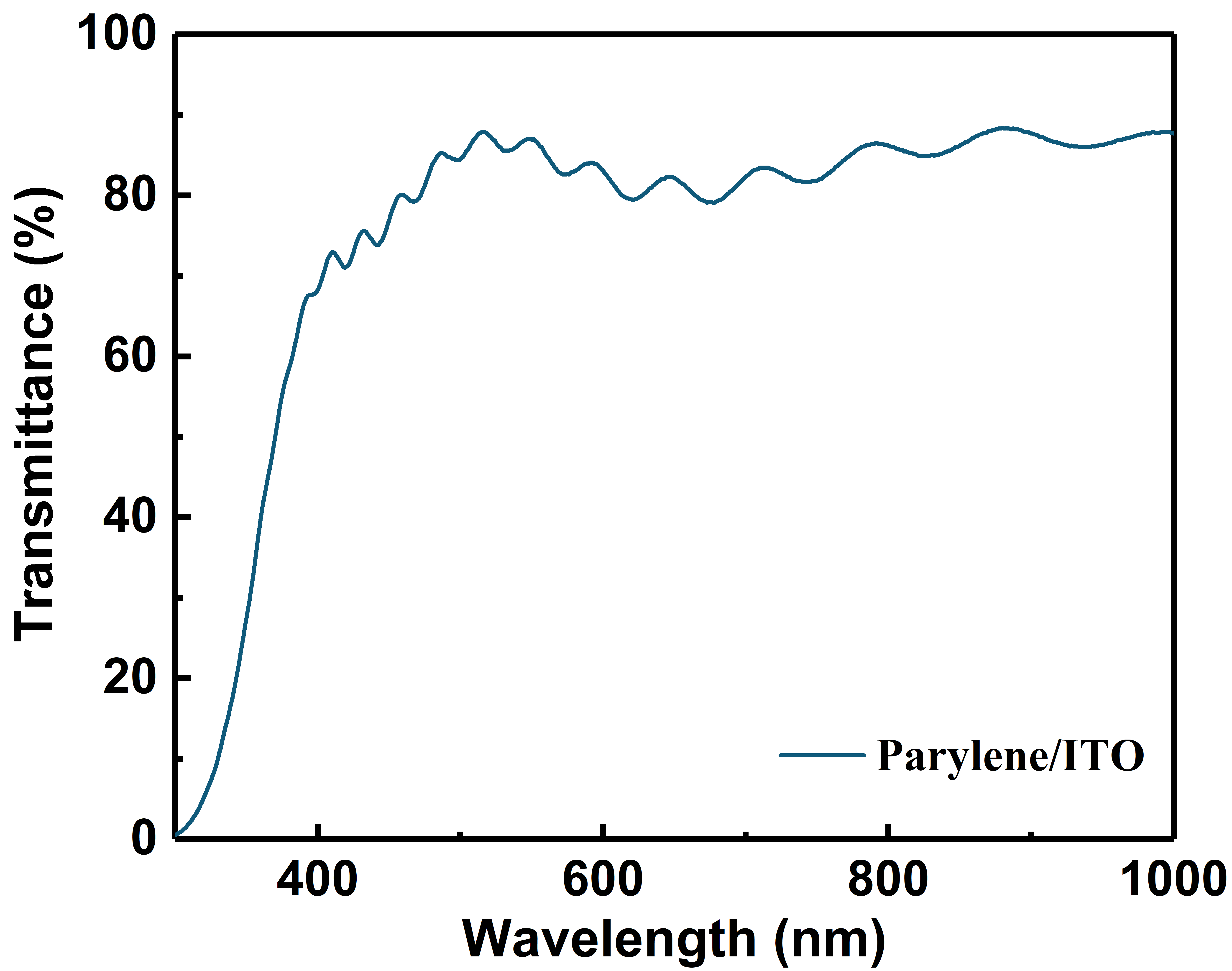
**

**Figure S29.** The transmittance of flexible parylene/ITO substrate.

**Table S1.** The electrical conductivities of ZnO electron transport layer with different In doping contents.

| Structure | σ_0_ [S m^-1^] |
| --- | --- |
| ITO/ZnO/Ag | 1.39×10^-4^ |
| ITO/IZO(1%)/Ag | 1.80×10^-4^ |
| ITO/IZO(3%)/Ag | 2.04×10^-4^ |
| ITO/IZO(5%)/Ag | 2.22×10^-4^ |
| ITO/IZO(7%)/Ag | 2.34×10^-4^ |
| ITO/IZO(9%)/Ag | 1.98×10^-4^ |

**Table S2.** Analytical data of GIWAXS results for ZnO and IZO films.

| ETL | (001) Out of plane | | | |  | | (001) In plane | | | | |  |
| --- | --- | --- | --- | --- | --- | --- | --- | --- | --- | --- | --- | --- |
|  | Q  [Å^-1^] | d  [Å] | FWHM  [Å^-1^] | CCL  [Å] | |  | | Q  [Å^-1^] | d  [Å] | FWHM  [Å^-1^] | CCL  [Å] | |
| ZnO | 1.51 | 4.16 | 0.324 | 17.45 | |  | | 1.52 | 4.13 | 0.401 | 14.10 | |
| IZO | 1.51 | 4.16 | 0.329 | 17.18 | |  | | 1.52 | 4.13 | 0.406 | 13.93 | |

**Table S3.** Photovoltaic parameters of PM6:L8-BO OSCs with different In doping content.

| Active layer^a)^ | ETL | *V*_oc_ [V] | *J*_sc_ [mA cm^-2^] | Calc *J*_sc_  [mA cm^-2^]^b)^ | FF [%] | PCE [%] |
| --- | --- | --- | --- | --- | --- | --- |
| PM6:L8-BO | ZnO | 0.869  (0.864±0.005) | 24.95  (24.93±0.02) | 24.03 | 76.88  (76.04±0.84) | 16.64  (16.38±0.26) |
|  | IZO(1%) | 0.867  (0.864±0.003) | 25.37  (25.21±0.15) | 24.20 | 76.71  (76.00±0.71) | 16.80  (16.57±0.23) |
|  | IZO(3%) | 0.865  (0.862±0.003) | 25.77  (25.68±0.09) | 24.82 | 76.64  (76.08±0.56) | 17.05  (16.84±0.21) |
|  | IZO(5%) | 0.863  (0.858±0.005) | 26.34  (26.32±0.04) | 25.50 | 77.17  (76.44±0.63) | 17.46  (17.27±0.19) |
|  | IZO(7%) | 0.861  (0.857±0.004) | 26.40  (26.27±0.13) | 25.35 | 76.59  (75.95±0.64) | 17.22  (17.11±0.11) |
|  | IZO(9%) | 0.829  (0.802±0.027) | 27.56  (26.98±0.58) | 25.31 | 52.45  (46.38±6.07) | 11.48  (10.05±1.43) |

^a)^ The average results from 8 devices; ^b)^ Calculated current densities from EQE curves.

**Table S4.** Photovoltaic parameters of PM6:L8-BO OSCs with different ETL thicknesses.

| ETL^a)^ | Thickness [nm] | *V*_oc_ [V] | *J*_sc_ [mA cm^-2^] | Calc *J*_sc_  [mA cm^-2^]^b)^ | FF [%] | PCE [%] |
| --- | --- | --- | --- | --- | --- | --- |
| IZO | 13 | 0.861  (0.858±0.003) | 26.65  (26.55±0.10) | 25.98 | 75.29  (74.55±0.74) | 17.20  (16.99±0.21) |
|  | 21 | 0.859  (0.857±0.002) | 26.80  (26.65±0.15) | 25.92 | 75.89  (75.32±0.57) | 17.43  (17.22±0.21) |
|  | 30 | 0.859  (0.856±0.003) | 26.72  (26.45±0.27) | 25.80 | 76.00  (75.50±0.50) | 17.25  (17.10±0.15) |

^a)^ The average results from 8 devices; ^b)^ Calculated current densities from EQE curves.

**Table S5.** Photovoltaic parameters of PM6:L8-BO OSCs with different ZnO annealing temperatures.

| ETL^a)^ | Annealing temperature [℃] | *V*_oc_ [V] | *J*_sc_ [mA cm^-2^] | Calc *J*_sc_  [mA cm^-2^]^b)^ | FF [%] | PCE [%] |
| --- | --- | --- | --- | --- | --- | --- |
| ZnO | 100 | 0.847  (0.845±0.002) | 24.10  (24.04±0.06) | 8.79 | 62.09  (61.32±0.77) | 12.63  (12.46±0.17) |
|  | 120 | 0.862  (0.859±0.003) | 24.80  (24.74±0.06) | 23.92 | 77.34  (76.39±0.95) | 16.49  (16.24±0.25) |
|  | 140 | 0.864  (0.862±0.002) | 25.13  (24.97±0.16) | 24.07 | 77.87  (77.46±0.41) | 16.85  (16.67±0.18) |
|  | 175 | 0.862  (0.860±0.002) | 25.41  (25.34±0.07) | 24.46 | 78.30  (77.30±0.10) | 17.11  (16.85±0.26) |
|  | 200 | 0.864  (0.861±0.003) | 25.85  (25.75±0.10) | 24.76 | 77.75  (77.19±0.56) | 17.28  (17.12±0.16) |

^a)^ The average results from 8 devices; ^b)^ Calculated current densities from EQE curves.

**Table S6.** Photovoltaic parameters of PM6:L8-BO OSCs with different IZO annealing temperatures.

| ETL^a)^ | Annealing temperature [℃] | *V*_oc_ [V] | *J*_sc_ [mA cm^-2^] | Calc *J*_sc_  [mA cm^-2^]^b)^ | FF [%] | PCE [%] |
| --- | --- | --- | --- | --- | --- | --- |
| IZO | 100 | 0.861  (0.831±0.030) | 2.86  (2.48±0.38) | 3.91 | 16.71  (15.50±1.21) | 0.40  (0.33±0.07) |
|  | 120 | 0.865  (0.863±0.002) | 26.27  (26.24±0.03) | 25.29 | 76.66  (76.17±0.49) | 17.43  (17.26±0.17) |
|  | 140 | 0.868  (0.864±0.004) | 26.50  (26.45±0.05) | 25.49 | 77.24  (76.96±0.28) | 17.70  (17.59±0.11) |
|  | 175 | 0.861  (0.859±0.002) | 26.01  (25.95±0.06) | 25.12 | 76.42  (75.84±0.58) | 17.11  (16.92±0.19) |
|  | 200 | 0.860  (0.858±0.002) | 25.85  (25.76±0.09) | 24.82 | 76.18  (75.31±0.87) | 16.87  (16.65±0.22) |

^a)^ The average results from 8 devices; ^b)^ Calculated current densities from EQE curves.

**Table S7.** *J_sat_*, *P_diss_*, *P_coll_*, and electron mobilities of PM6:L8-BO OSCs prepared with ZnO or IZO ETL.

| ETL/Active layer | *J*_sat_ [mA cm^-2^] | *P*_diss_ [%] | *P*_coll_ [%] | Electron Mobility [*μ_e_*] [cm^2^ V^-1^ s^-1^] |
| --- | --- | --- | --- | --- |
| ZnO/PM6:L8-BO | 26.40 | 95.2% | 86.0% | 4.4×10^-4^ |
| IZO/PM6:L8-BO | 27.00 | 98.2% | 89.4% | 5.8×10^-4^ |

**Table S8.** Analytical data of GIWAXS results for PM6:L8-BO blend films on different ETLs.

| ETL | (010) Out of plane | | | |  | | (100) In plane | | | | |  |
| --- | --- | --- | --- | --- | --- | --- | --- | --- | --- | --- | --- | --- |
|  | Q  [Å^-1^] | d  [Å] | FWHM  [Å^-1^] | CCL  [Å] | |  | | Q  [Å^-1^] | d  [Å] | FWHM  [Å^-1^] | CCL  [Å] | |
| ZnO | 1.73 | 3.63 | 0.355 | 15.92 | |  | | 0.30 | 20.94 | 0.078 | 72.49 | |
| IZO | 1.73 | 3.63 | 0.352 | 16.06 | |  | | 0.30 | 20.94 | 0.076 | 74.40 | |

**Table S9.** *J_sat_*, *P_diss_*, *P_coll_*, and electron mobilities of PM6:L8-BO OSCs on different ETLs.

| ETL/Active layer | *J*_sat_ [mA cm^-2^] | *P*_diss_ [%] | *P*_coll_ [%] | Electron Mobility [*μ_e_*] [cm^2^ V^-1^ s^-1^] |
| --- | --- | --- | --- | --- |
| ZnO/PM6:L8-BO | 26.40 | 95.2% | 86.0% | 4.4×10^-4^ |
| IZO/PM6:L8-BO | 27.00 | 98.2% | 89.4% | 5.8×10^-4^ |

**Table S10.** Summary of the efficiency of Inverted structure device.

| Rigid inverted OSCs | | | | |  |  |
| --- | --- | --- | --- | --- | --- | --- |
| Year | Structures | BHJ | PCE [%] | Ref. |  |  |
| 2022 | ITO/ZnO/PET/BHJ/MoO_3_/Ag | PM6:Y6 | 15.03 | ^[1]^ |  |  |
| 2021 | ITO/ZnO/PET/BHJ/MoO_3_/Ag | PM6:Y6 | 16.46 | ^[2]^ |  |  |
| 2022 | ITO/SnO_2_/PAS/BHJ/MoO_3_/Ag | PM6:Y6 | 16.37 | ^[3]^ |  |  |
| 2020 | ITO/OSiNDs/BHJ/MoO_3_/Ag | PM6:Y6:PC_71_BM | 17.15 | ^[4]^ |  |  |
| 2020 | ITO/ZnO/BHJ/MoO_3_/Ag | PM6:Y6:PC_71_BM | 16.63 | ^[4]^ |  |  |
| 2022 | ITO/CD/BHJ/MoO_3_/Ag | PM6:Y6:PC_71_BM | 16.80 | ^[5]^ |  |  |
| 2022 | ITO/CD/BHJ/MoO_3_/Ag | PM6:BTP-eC9 | 17.35 | ^[5]^ |  |  |
| 2022 | ITO/SnO_2_/1-DPAQ/BHJ/MoO_3_/Ag | PM6:BTP-eC9 | 17.70 | ^[6]^ |  |  |
| 2022 | ITO/SnO_2_/1-DPAQ/BHJ/MoO_3_/Ag | PM6:PB2F:BTP-eC9 | 18.10 | ^[6]^ |  |  |
| 2021 | ITO/ZnO:Zr /BHJ/MoO_3_/Ag | PM6:BTP-eC9 | 17.70 | ^[7]^ |  |  |
| 2021 | ITO/PA-ZnO /BHJ/MoO_3_/Ag | PM6:BTP-eC9 | 17.60 | ^[8]^ |  |  |
| 2022 | ITO/SnO_2_/PAS/BHJ/MoO_3_/Ag | PM6:BTP-eC9 | 17.12 | ^[3]^ |  |  |
| 2022 | ITO/ZnO/BHJ/MoO_3_/Ag | PM6:N3 | 15.00 | ^[1]^ |  |  |
| 2022 | ITO/ZnO/BHJ/MoO_3_/Ag | PM6:ITIC | 10.28 | ^[1]^ |  |  |
| 2022 | ITO/ZnO/PET/BHJ/MoO_3_/Ag | PM6:L8-BO | 17.02 | ^[1]^ |  |  |
| 2022 | ITO/ZnO/BHJ/MoO_3_/Ag | PM6:IT-4F | 11.18 | ^[1]^ |  |  |
| 2019 | ITO/ZnO:PFN-Br/BHJ/MoO_3_/Ag | PBDB-TF:IT-4F | 13.82 | ^[9]^ |  |  |
| 2018 | ITO/PVP/BHJ/MoO_3_/Ag | PBDB-TF:IT-4F | 13.30 | ^[10]^ |  |  |
| 2014 | ITO/ZnO/CsSt/BHJ/MoO_3_/Ag | PTB7:PC_71_BM | 8.69 | ^[11]^ |  |  |
| 2019 | ITO/PFN/BHJ/DPA2T/MoO_3_/Ag | PTB7:PC_71_BM | 9.32 | ^[12]^ |  |  |
| 2018 | ITO/PEI/BHJ/PEDOT:PSS/MoO_3_/Ag | PTB7:PC_71_BM | 9.54 | ^[13]^ |  |  |
| 2017 | ITO/TiO_2_/BHJ/CuBr_2_/MoO_3_/Ag | PTB7:PC_71_BM | 9.54 | ^[14]^ |  |  |
| 2017 | ITO/PEIE/BHJ/ /MoO_3_/PEIE/Ag | PTB7-Th:PC_71_BM | 8.42 | ^[15]^ |  |  |
| 2016 | ITO/ZnO-NPs/BHJ/PEDOT:PSS/MoO_3_/Ag | CdSe:P3HT | 1.53 | ^[16]^ |  |  |
| 2020 | ITO/PEIE/BHJ/CPB:MoO_3_/Ag | P3HT:PCBM | 2.38 | ^[17]^ |  |  |
| 2012 | ITO/ZnO/BHJ/MTDATA/MoO_3_/Ag | PDTS-BTD:PC_71_BM | 6.45 | ^[18]^ |  |  |
| 2022 | ITO/AZO/BHJ/2PACz/MoO_3_/Ag | PM6:Y6 | 16.49 | ^[19]^ |  |  |
| 2022 | ITO/AZO/BHJ/2PACz/MoO_3_/Ag | PM6: Y6-BO | 17.10 | ^[19]^ |  |  |
| 2022 | ITO/AZO/BHJ/2PACz/MoO_3_/Ag | PM6:L8-BO:Y6-BO | 18.49 | ^[19]^ |  |  |
| 2022 | ITO/Li-ZnO/BHJ/MoO_3_/Ag | PM6:Y6 | 13.66 | ^[20]^ |  |  |
| 2022 | ITO/ZnO/CA/BHJ/MoO_3_/Ag | PM6:Y6 | 16.56 | ^[21]^ |  |  |
| 2022 | ITO/ZnO/CA/BHJ/MoO_3_/Ag | PM6:L8-BO | 18.22 | ^[21]^ |  |  |
| 2020 | ITO/PEI-Zn/BHJ/MoO_3_/Ag | PBDBT-2F:IT-4F | 12.80 | ^[22]^ |  |  |
| 2022 | ITO/ZnO/PEIE-PAC/BHJ/MoO_3_/Ag | PM6:Y6 | 16.47 | ^[23]^ |  |  |
| 2022 | ITO/ZnO/NMA/BHJ/MoO_3_/Ag | D18:N3 | 18.20 | ^[24]^ |  |  |
| 2021 | ITO/ZnO:Zr/ BHJ/ MoO_3_/Ag | PM6:Y6:PC_71_BM | 17.20 | ^[25]^ |  |  |
| 2022 | ITO/ZnO/BA/BHJ/MoO_3_/Al | PBDB-TF:HDO-4Cl:BTP-eC9 | 18.40 | ^[26]^ |  |  |
| 2022 | ITO/ZnO/BA/BHJ/MoO_3_/Al | PBDB-TF: BTP-eC9 | 17.17 | ^[26]^ |  |  |
| 2022 | ITO/(ZnO)_EA-free_/BHJ/MoO_3_/Ag | PM6:L8-BO | 17.57 | ^[27]^ |  |  |
| 2021 | ITO/ZnO/BHJ/MoO_3_/Ag | PM6:L8-BO | 18.05 | ^[28]^ |  |  |
| 2022 | ITO/ZnO/BHJ/MoO_3_/Ag | PM6:BTC:L8-BO | 18.41 | ^[29]^ |  |  |
| 2019 | ITO/ZnO/BHJ/MoO_3_/Ag | PM6:Y6 | 15.70 | ^[30]^ |  |  |
| 2022 | ITO/ZnO/BHJ/MoO_3_/Ag | PM6:BTP-BO-4F:DBDMF | 17.20 | ^[31]^ |  |  |
| 2018 | ITO/In_2_O_3_/PEIE/BHJ/MoO_3_/Ag | PBDTTT-EFT:IEICO-4F | 11.50 | ^[32]^ |  |  |
| 2023 | ITO/Ir/IrO_x_/BHJ/MoO_x_/Al | PM6:Y6:PC_71_BM | 16.19 | ^[33]^ |  |  |
| 2023 | ITO/ZnO/PAA/BHJ/MoO_3_/Ag | PM6:BTP-4F-C5-16 | 18.00 | ^[34]^ |  |  |
| 2023 | ITO/ZnO/NMA/BHJ/MoO_x_/Ag | PM6:CH7 | 17.49 | ^[35]^ |  |  |
| 2023 | ITO/IZO/BHJ/ MoO_3_/Ag | PM6:L8-BO | 17.81 | This work | |  |
| 2023 | ITO/IZO/BHJ/ MoO_3_/Ag | PM6:L8-BO:BTP-eC9 | 18.11 | This work | |  |
| Inverted Ultrathin flexible OSCs | | | | | | |
| Year | Structures | BHJ | PCE [%] | Ref. | |  |
| 2020 | Parylene/SU8/ITO/ZnO/BHJ/MoO_3_/Ag | PBDTTT-OFT:IEICO-4F:PC_71_BM | 13.00 | ^[36]^ | |  |
| 2023 | PI-ZnO/Ag NWs/ZnO/BHJ/MoO_3_/Ag | PM6:BTP-4Cl | 13.55 | ^[37]^ | |  |
| 2022 | HB-PI/Ag NWs/ZnO/BHJ/MoO_3_/Ag | PM6:BTP-4Cl | 13.52 | ^[38]^ | |  |
| 2018 | Parylene/ITO/ZnO NP/BHJ/MoO_3_/Ag | PBDTTT-OFT:PC_71_BM | 10.67 | ^[39]^ | |  |
| 2021 | Parylene/SU8/ITO/ZnO/BHJ/PENOT:PSS/Ag | PBDTTT-OFT:IEICO-4F | 10.40 | ^[40]^ | |  |
| 2017 | Parylene/ITO/ZnO/BHJ/MoO_3_/Ag | PNTz4T:PC_71_BM | 7.90 | ^[41]^ | |  |
| 2021 | Parylene/ITO/ZnO/BHJ/MoO_3_/Ag | PBDTTT-OFT:PC_71_BM | 10.60 | ^[42]^ | |  |
| 2020 | PEN/Ag NWs/PEI-Zn/BHJ//MoO_3_/Ag | PM6:IT-4F | 10.40 | ^[22]^ | |  |
| 2020 | PEN/PH1000/PEI-Zn/BHJ//MoO_3_/Ag | PM6:IT-4F | 12.90 | ^[22]^ | |  |
| 2020 | PEN/Ag NWs/PEI-Zn/BHJ//MoO_3_/Ag | PM6:Y6 | 12.30 | ^[22]^ | |  |
| 2020 | PEN/PH1000/PEI-Zn/BHJ//MoO_3_/Ag | PM6:Y6 | 15.03 | ^[22]^ | |  |
| 2019 | PI/ITO/ZnO/BHJ/MoO_3_/Ag | PTzNTz-BOBO:PC_71_BM | 9.30 | ^[43]^ | |  |
| 2022 | tPI/ITO/PEI-Zn/BHJ/MoO_3_/Ag | PM6:Y6 | 15.80 | ^[44]^ | |  |
| 2023 | Parylene/ITO/IZO/BHJ/MoO_3_/Ag | PM6:L8-BO:BTP-eC9 | 17.01 | This work | |  |

[1] B. Liu, X. Su, Y. Lin, Z. Li, L. Yan, Y. Han, Q. Luo, J. Fang, S. Yang, H. Tan, C. Q. Ma, *Adv. Sci.* **2022**, *9*, 2104588.

[2] Y. Han, H. Dong, W. Pan, B. Liu, X. Chen, R. Huang, Z. Li, F. Li, Q. Luo, J. Zhang, Z. Wei, C. Q. Ma, *ACS Appl. Mater. Interfaces* **2021**, *13*, 17869.

[3] H. Gao, X. Wei, R. Yu, F. Y. Cao, Y. Gong, Z. Ma, Y. J. Cheng, C. S. Hsu, Z. a. Tan, *Adv. Opt. Mater.* **2022**, *10*, 2102031.

[4] M. Cui, D. Li, X. Du, N. Li, Q. Rong, N. Li, L. Shui, G. Zhou, X. Wang, C. J. Brabec, L. Nian, *Adv. Mater.* **2020**, *32*, 2002973.

[5] Y. Dong, R. Yu, B. Zhao, Y. Gong, H. Jia, Z. Ma, H. Gao, Z. Tan, *ACS Appl. Mater. Interfaces* **2022**, *14*, 1280.

[6] R. Yu, X. Wei, G. Wu, T. Zhang, Y. Gong, B. Zhao, J. Hou, C. Yang, Z. a. Tan, *Energy Environ. Sci.* **2022**, *15*, 822.

[7] X. Song, G. Liu, P. Sun, Y. Liu, W. Zhu, *J Phys Chem Lett* **2021**, *12*, 10616.

[8] X. Liu, Z. Zheng, J. Wang, Y. Wang, B. Xu, S. Zhang, J. Hou, *Adv. Mater.* **2022**, *34*, 2106453.

[9] Z. Zheng, S. Zhang, J. Wang, J. Zhang, D. Zhang, Y. Zhang, Z. Wei, Z. Tang, J. Hou, H. Zhou, *J. Mater. Chem. A* **2019**, *7*, 3570.

[10] B. Yang, S. Zhang, S. Li, H. Yao, W. Li, J. Hou, *Adv. Mater.* **2019**, *31*, 1804657.

[11] G. Wang, T. Jiu, G. Tang, J. Li, P. Li, X. Song, F. Lu, J. Fang, *ACS Sustain. Chem. Eng.* **2014**, *2*, 1331.

[12] Y. Jiang, H. Peng, R. Mai, Y. Meng, Q. Rong, C. Cabanetos, L. Nian, J. Roncali, G. Zhou, J. Liu, J. Gao, *Org. Electron.* **2019**, *68*, 200.

[13] Z. Li, C. Liu, J. Guo, X. Zhang, Y. Zhou, L. Shen, W. Guo, *Sol Energy* **2018**, *171*, 8.

[14] Z. Li, W. Guo, C. Liu, X. Zhang, S. Li, J. Guo, L. Zhang, *Phys. Chem. Chem. Phys.* **2017**, *19*, 20839.

[15] D. Qin, H. Cao, C. Yan, S.-S. Meng, J.-X. Tang, X. Zhan, *J. Mater. Chem. A* **2017**, *5*, 25385.

[16] L. Zhu, B. J. Richardson, Q. Yu, *Phys. Chem. Chem. Phys.* **2016**, *18*, 3463.

[17] C. Chen, S. Jin, J. Zhang, Q. Yang, D. Qin, *Thin Solid Films* **2020**, *697*.

[18] J. Subbiah, C. M. Amb, I. Irfan, Y. Gao, J. R. Reynolds, F. So, *ACS Appl. Mater. Interfaces* **2012**, *4*, 866.

[19] Q. Huang, J. Jing, K. Zhang, Y. Chen, A. Song, Z. Liu, F. Huang, *J. Mater. Chem. A* **2022**, *10*, 23973.

[20] J. Wang, H. Pan, X. Xu, H. Jin, W. Ma, S. Xiong, Q. Bao, Z. Tang, Z. Ma, *ACS Appl. Mater. Interfaces* **2022**, *14*, 12450.

[21] S. Yang, H. Yu, *Chem. Eng. J.* **2023**, *452*, 139658.

[22] F. Qin, W. Wang, L. Sun, X. Jiang, L. Hu, S. Xiong, T. Liu, X. Dong, J. Li, Y. Jiang, J. Hou, K. Fukuda, T. Someya, Y. Zhou, *Nat. Commun.* **2020**, *11*, 4508.

[23] H. Zheng, D. Zhou, L. Hu, Z. Xu, H. Xu, Y. Zhang, Y. Tong, B. Hu, Z. Li, L. Chen, *Sol. RRL* **2022**, *6*, 2200871.

[24] S. Li, Q. Fu, L. Meng, X. Wan, L. Ding, G. Lu, G. Lu, Z. Yao, C. Li, Y. Chen, *Angew. Chem. Int. Ed.* **2022**, *61*, 202207397.

[25] X. Song, G. Liu, W. Gao, Y. Di, Y. Yang, F. Li, S. Zhou, J. Zhang, *Small* **2021**, *17*, 2006387.

[26] Y. Wang, Z. Zheng, J. Wang, X. Liu, J. Ren, C. An, S. Zhang, J. Hou, *Adv. Mater.* **2022**, *35*, 2208305.

[27] P. Jiang, J. Chen, F. Qin, T. Liu, S. Xiong, W. Wang, C. Xie, X. Lu, Y. Jiang, H. Han, Y. Zhou, *Angew. Chem. Int. Ed.* **2022**, *61*, 202208815.

[28] C. Li, J. Zhou, J. Song, J. Xu, H. Zhang, X. Zhang, J. Guo, L. Zhu, D. Wei, G. Han, J. Min, Y. Zhang, Z. Xie, Y. Yi, H. Yan, F. Gao, F. Liu, Y. Sun, *Nat. Energy* **2021**, *6*, 605.

[29] C. Zhang, J. Li, L. Ji, H. Hu, G. Li, K. Wang, *J. Mater. Chem. A* **2022**, *10*, 22812.

[30] J. Yuan, Y. Zhang, L. Zhou, G. Zhang, H.-L. Yip, T.-K. Lau, X. Lu, C. Zhu, H. Peng, P. A. Johnson, M. Leclerc, Y. Cao, J. Ulanski, Y. Li, Y. Zou, *Joule* **2019**, *3*, 1140.

[31] S.-C. Huang, M. R. Busireddy, H.-F. Chang, I. C. Pan, C.-H. Ho, C.-W. Ko, Y.-W. Tsai, J.-M. Lin, J.-T. Chen, C.-S. Hsu, *Sol. RRL* **2022**, *6*, 2200805.

[32] W. Huang, B. Zhu, S. Y. Chang, S. Zhu, P. Cheng, Y. T. Hsieh, L. Meng, R. Wang, C. Wang, C. Zhu, C. McNeill, M. Wang, Y. Yang, *Nano Lett.* **2018**, *18*, 5805.

[33] Y. Li, B. Huang, X. Zhang, J. Ding, Y. Zhang, L. Xiao, B. Wang, Q. Cheng, G. Huang, H. Zhang, Y. Yang, X. Qi, Q. Zheng, Y. Zhang, X. Qiu, M. Liang, H. Zhou, *Nat. Commun.* **2023**, *14*, 1241.

[34] Y. Liu, L. Wang, B. Zhou, Y. Fu, D. Li, C. Guo, H. Wang, C. Chen, J. Cai, X. Zhang, W. Sun, D. Liu, W. Li, T. Wang, *ACS Materials Letters* **2022**, *5*, 321.

[35] S. Zhang, H. Chen, P. Wang, S. Li, Z. Li, Y. Huang, J. Liu, Z. Yao, C. Li, X. Wan, Y. Chen, *Solar RRL* **2023**, *7*, 2300029.

[36] W. Huang, Z. Jiang, K. Fukuda, X. Jiao, C. R. McNeill, T. Yokota, T. Someya, *Joule* **2020**, *4*, 128.

[37] Y. Wang, Q. Chen, G. Zhang, Y. Wang, Z. Zhang, J. Fang, C. Zhao, W. Li, *Chem. Eng. J.* **2023**, *451*, 138612.

[38] Y. Wang, Q. Chen, Y. Wang, G. Zhang, Z. Zhang, J. Fang, C. Zhao, W. Li, *Macromol. Rapid Commun.* **2022**, *43*, 2200432.

[39] S. Park, S. W. Heo, W. Lee, D. Inoue, Z. Jiang, K. Yu, H. Jinno, D. Hashizume, M. Sekino, T. Yokota, K. Fukuda, K. Tajima, T. Someya, *Nature* **2018**, *561*, 516.

[40] S. I. Rich, S. Lee, K. Fukuda, T. Someya, *Adv. Mater.* **2022**, *34*, 2106683.

[41] H. Jinno, K. Fukuda, X. Xu, S. Park, Y. Suzuki, M. Koizumi, T. Yokota, I. Osaka, K. Takimiya, T. Someya, *Nat. Energy* **2017**, *2*, 780.

[42] H. Jinno, T. Yokota, M. Koizumi, W. Yukita, M. Saito, I. Osaka, K. Fukuda, T. Someya, *Nat. Commun.* **2021**, *12*, 2234.

[43] H. Kimura, K. Fukuda, H. Jinno, S. Park, M. Saito, I. Osaka, K. Takimiya, S. Umezu, T. Someya, *Adv. Mater.* **2019**, *31*, 1808033.

[44] S. Xiong, K. Fukuda, S. Lee, K. Nakano, X. Dong, T. Yokota, K. Tajima, Y. Zhou, T. Someya, *Adv. Sci.* **2022**, *9*, 2105288.

[45] X. Zheng, L. Zuo, K. Yan, S. Shan, T. Chen, G. Ding, B. Xu, X. Yang, J. Hou, M. Shi, H. Chen, *Energy Environ. Sci.* **2023**, *16*, 2284.
